# Supplementary material for: Nitrocellulose redox permanganometry: A simple method for reductive capacity assessment
Source: MethodsX. 2021 Dec 22;9:101611. doi: 10.1016/j.mex.2021.101611 (PMC8718987; doi:10.1016/j.mex.2021.101611)
Supplement: Supplementary file 1 [file mmc1.docx]

**Supplementary Material for „Nitrocellulose redox permanganometry: a simple method for reductive capacity assessment“**

Contents

[Supplementary material 1: A step-by-step Nitrocellulose redox permanganometry (NRP) and HistoNRP protocols 3](#_Toc49964296)

[A step-by-step Nitrocellulose Redox Permanganometry (NRP) protocol for liquid samples 3](#_Toc49964297)

[A step-by-step HistoNRP protocol for cryosections 9](#_Toc49964298)

[A step-by-step HistoNRP protocol for formalin-fixed paraffin-embedded (FFPE) tissue samples 15](#_Toc49964299)

[Supplementary material 2: Nitrocellulose redox permanganometry (NRP) linearity, precision and accuracy validation tests 23](#_Toc49964300)

[Supplementary material 3: Nitrocellulose redox permanganometry (NRP) ascorbic acid heating time and temperature response validation experiments 32](#_Toc49964301)

[Supplementary material 4: Nitrocellulose redox permanganometry (NRP) membrane stability analysis 36](#_Toc49964302)

[Supplementary material 5: Comparison of Nitrocellulose redox permanganometry (NRP) digitalization techniques 40](#_Toc49964303)

[Supplementary material 6: An explanation of the computational analysis of Nitrocellulose redox permanganometry (NRP) 44](#_Toc49964304)

[Supplementary material 7: Additional replications of ORP-NRP experiments from Fig 1E and Fig 1F 50](#_Toc49964305)

[Supplementary material 8: A detailed explanation of the origin of animal tissue used in the Nitrocellulose redox permanganometry (NRP) proof of concept experiments 51](#_Toc49964306)

[Experiment 1: 52](#_Toc49964307)

[Experiment 2: 54](#_Toc49964308)

[Experiment 3: 57](#_Toc49964309)

[Supplementary material 9: Nitrocellulose redox permanganometry (NRP) protein concentration-based correction analysis 59](#_Toc49964310)

[Supplementary material 10: HistoNRP adaptation for the analysis of formalin-fixed paraffin-embedded (FFPE) tissue sections 62](#_Toc49964311)

[Supplementary material 11: A detailed explanation of the HistoNRP demonstration analysis illustrated in the Fig 2 of the Main text 67](#_Toc49964312)

[References: 69](#_Toc49964313)

Supplementary material 1: A step-by-step Nitrocellulose redox permanganometry (NRP) and HistoNRP protocols

## A step-by-step Nitrocellulose Redox Permanganometry (NRP) protocol for liquid samples

Materials and reagents

- A piece of nitrocellulose membrane

- A small volume pipette

- Pipette tips

- KMnO_4_

- ddH_2_O

- Tweezers

- A Petri dish

- Glass beaker

- A stirring magnet

- Magnetic stirrer

NRP Protocol

1. **Prepare the KMnO_4_ working solution by dissolving 0.2 g of solid KMnO_4_ crystals in 20 mL of ddH_2_O (place the solution with a magnet on the magnetic stirrer until all crystals dissolve).**

**
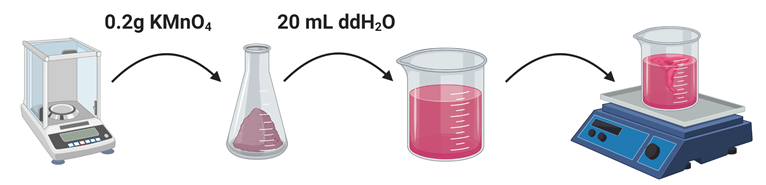
**

1. **Remove the protective cover from one side of the nitrocellulose membrane and place the membrane on a clean laboratory surface (leave the protective cover on the side of the membrane facing the laboratory surface).

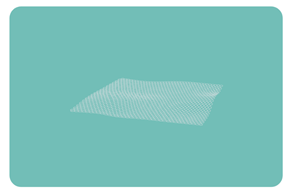
**
2. **Vortex samples.**

**
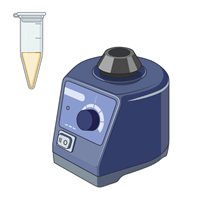
**

1. **Pipette 1 μL of the sample onto the nitrocellulose membrane, repeat this step with every sample you want to analyze.**

**
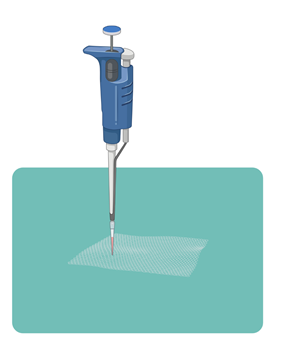
**

1. **Caution: Leave enough space between your samples.**

**
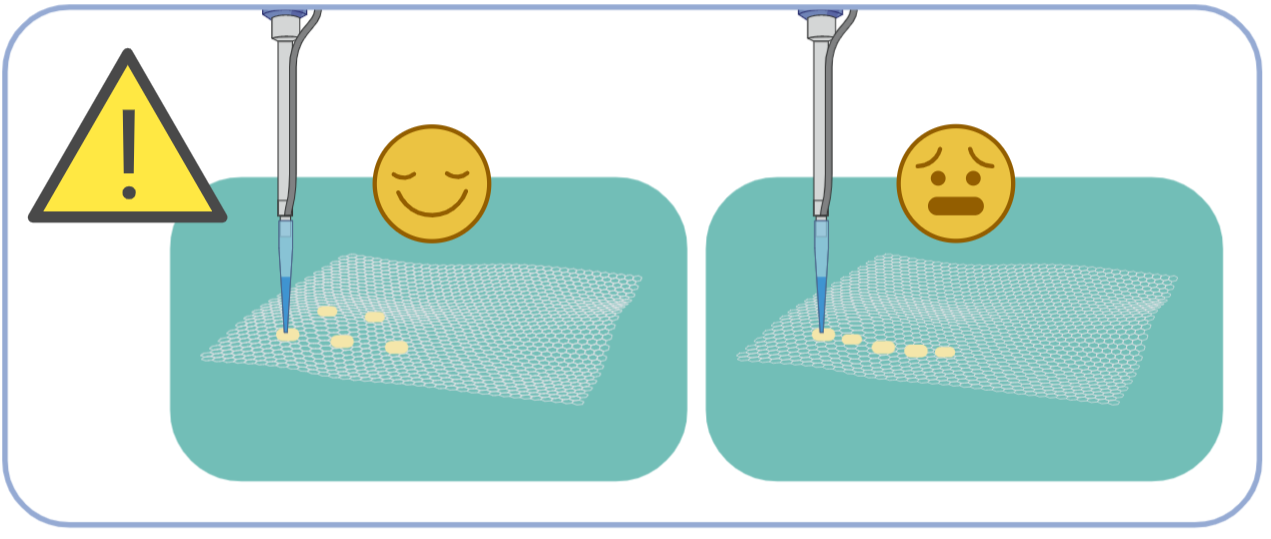
**

1. **Leave the nitrocellulose membrane to dry out.**

**
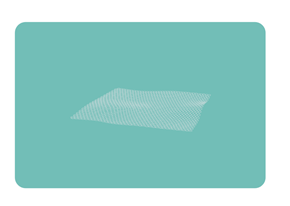
**

1. **Once dry, pick up the nitrocellulose membrane with tweezers and place it in the KMnO_4_ working solution. Wait for 30 seconds.**

**
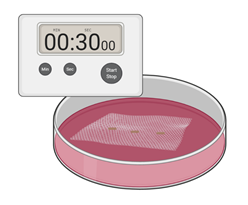
**

1. **Remove the nitrocellulose membrane from the KMnO_4_ working solution with tweezers and place it under running ddH_2_O to terminate the reaction and increase the contrast.**

**
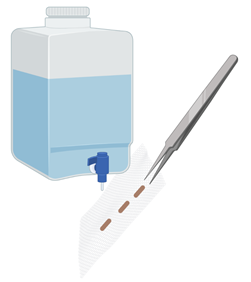
**

1. **Leave the nitrocellulose membrane to dry out.**

**
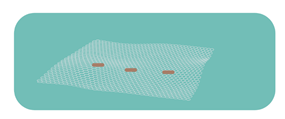
**

1. **Digitalize the membrane (with a cellphone, camera or an office scanner). Import the photo into Fiji (Fiji Is Just ImageJ) software.**

**
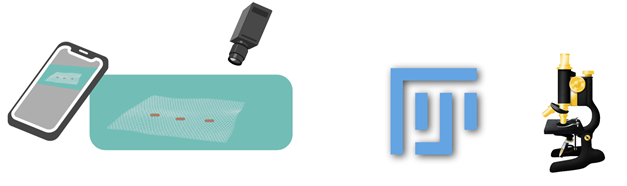
**

1. **Select the region of interest in Fiji by using the “Rectangle selection” tool*.***

**
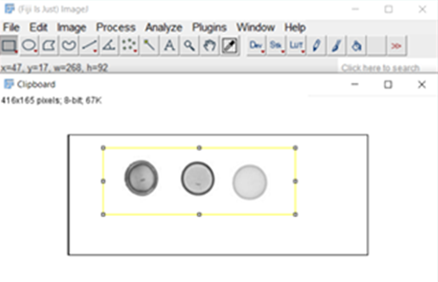
**

1. **Perform the Gel Analyzer plugin “Select First Lane” function (CTRL+1).

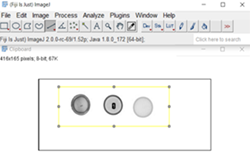
**
2. **Perform the Gel Analyzer plugin “Plot Lanes” function (CTRL+3).

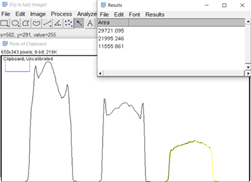
**
3. **Export values from the Fiji “Results” tab, and proceed to statistical analysis and visualization (greater values represent higher reductive (antioxidant) capacity).

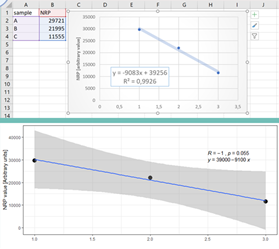
**

## A step-by-step HistoNRP protocol for cryosections

Materials and reagents

- A piece of nitrocellulose membrane

- Filter papers

- A pipette

- KMnO_4_

- ddH_2_O

- Phosphate buffered saline (1xPBS)

- Tweezers

- A Petri dish

- Glass beaker

- A glass plate

- A stirring magnet

- Magnetic stirrer

HistoNRP protocol for cryosections

1. **Prepare the KMnO_4_ working solution by dissolving 0.2 g of solid KMnO_4_ crystals in 20 mL of ddH_2_O (place the solution with a magnet on the magnetic stirrer until all crystals dissolve).**

**
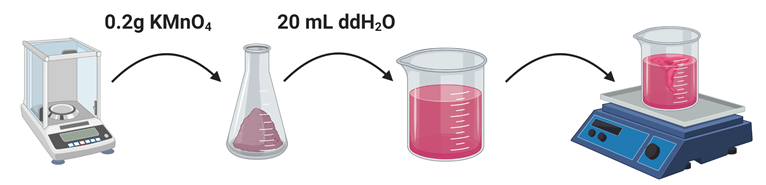
**

1. **Remove the frozen tissue samples from the freezer and use a cryostat to make cryosections of the tissue of interest. Place the sections directly onto the glass slides. Air-dry the samples at 37°C.**

**
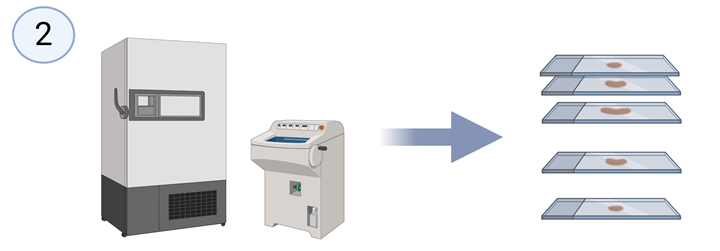
**

1. **Place the slides onto a clean laboratory surface and wet the tissue samples with PBS. Place a piece of nitrocellulose membrane onto the wetted slide. Place 3 filter papers on top of the membrane. Wet the filter papers with PBS.**

**
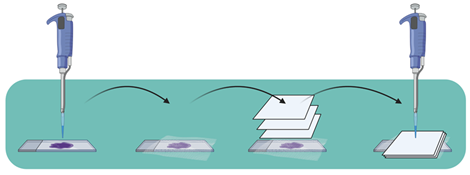
**

1. **Cover everything with a glass plate for even distribution of the weight and place a full beaker onto the glass plate to apply appropriate pressure (the optimal pressure should be determined by the end-user; in our case, it was 31.384 mmHg achieved by placing a beaker with 800 g of water on the glass plate). Leave the sample proteins to passively diffuse onto the membrane overnight at room temperature.**

**
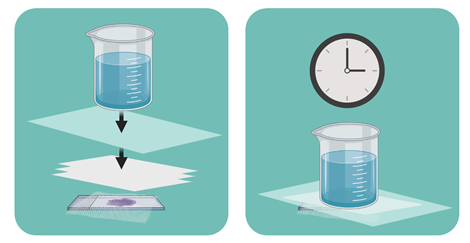
**

1. **Remove the beaker, glass plate, and 2 filter papers carefully. Wet the last filter paper with PBS and remove it carefully with tweezers. Add additional PBS and remove the membrane from the slide. Leave the membrane with printed proteins to dry out.**

**
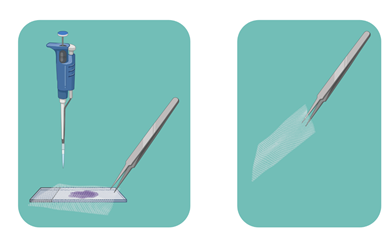
**

1. **Once dry, pick up the nitrocellulose membrane with tweezers and place it in the KMnO_4_ working solution for 30 seconds. Remove the nitrocellulose membrane from the KMnO_4_ working solution with tweezers and place it under running ddH_2_O to terminate the reaction and increase the contrast.

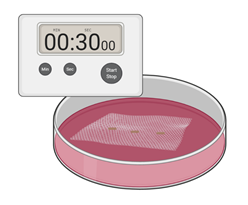

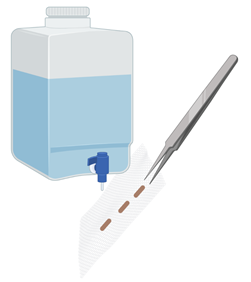
**
2. **Leave the nitrocellulose membrane to dry out and digitalize the membrane (with a cellphone, camera, or an office scanner). Import the photo into Fiji (Fiji is just ImageJ) software.**

**
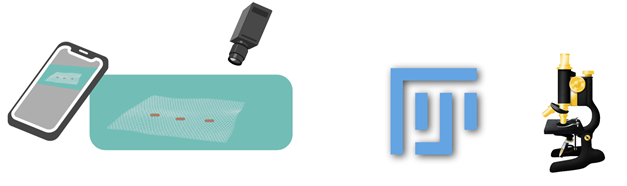
**

1. **Prepare the image for intensity analysis by 8-bit (Image>Type>8-bit) processing and appropriate inversion (Edit>Invert; “CTRL+shift+I”).**

**
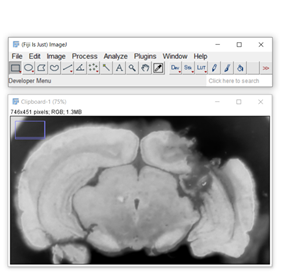
**

1. **Select the area of interest using the selection tool and measure pixel intensities by using the Fiji “Histogram” function (Analyze>Histogram; “CTRL+H”).**

**
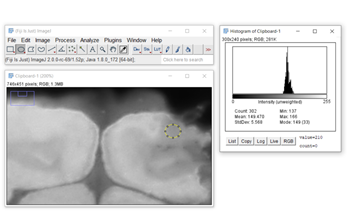
**

1. **Use the “List” option to export values from Fiji for further analysis.**

**
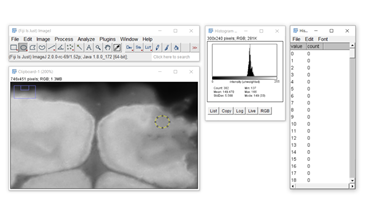
**

1. **An example of pixel intensity comparisons from the ipsilateral side following microdialysis probe-induced damage and the contralateral side of the brain used as a reference. A comparative analysis of 18 brain areas analyzed by density plot comparison done in the R software environment for statistical computing is further explained in the Main text.


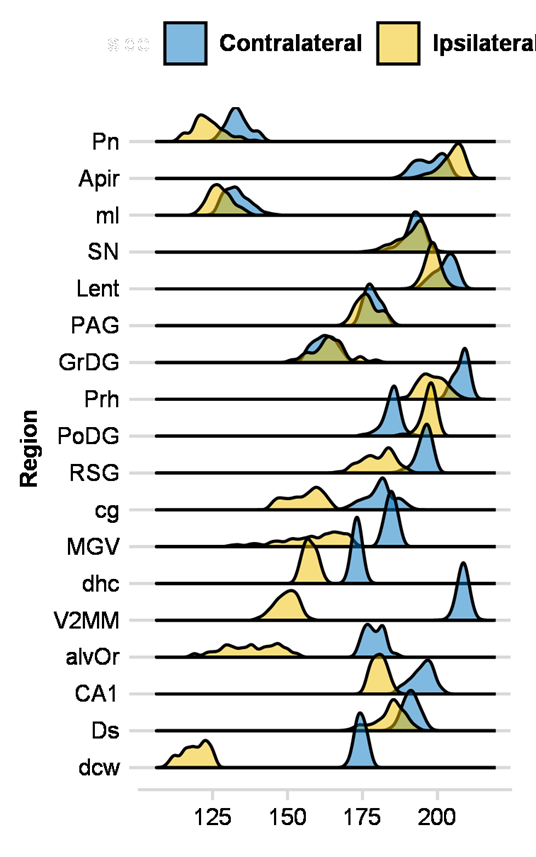
**

## A step-by-step HistoNRP protocol for formalin-fixed paraffin-embedded (FFPE) tissue samples

Materials and reagents

- A piece of nitrocellulose membrane

- Filter papers

- A pipette

- KMnO_4_

- ddH_2_O

- Phosphate buffered saline (1xPBS)

- Tweezers

- A Petri dish

- Glass beaker

- Two glass plates

- A stirring magnet

- Magnetic stirrer

- Laboratory heater

- Parafilm

- Equipment for FFPE tissue deparaffinization (Xylene, EtOH)

HistoNRP protocol for FFPE samples

1. **Prepare the KMnO_4_ working solution by dissolving 0.2 g of solid KMnO_4_ crystals in 20 mL of ddH_2_O (place the solution with a magnet on the magnetic stirrer until all crystals dissolve).**

**
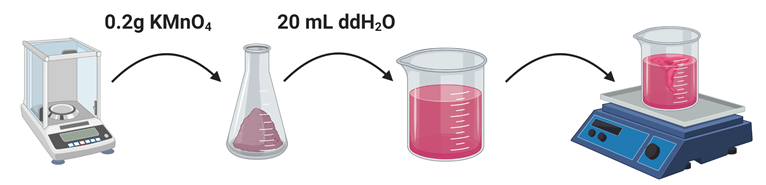
**

1. **Cut the FFPE tissue on the microtome and mount the sections on histological slides.**

**
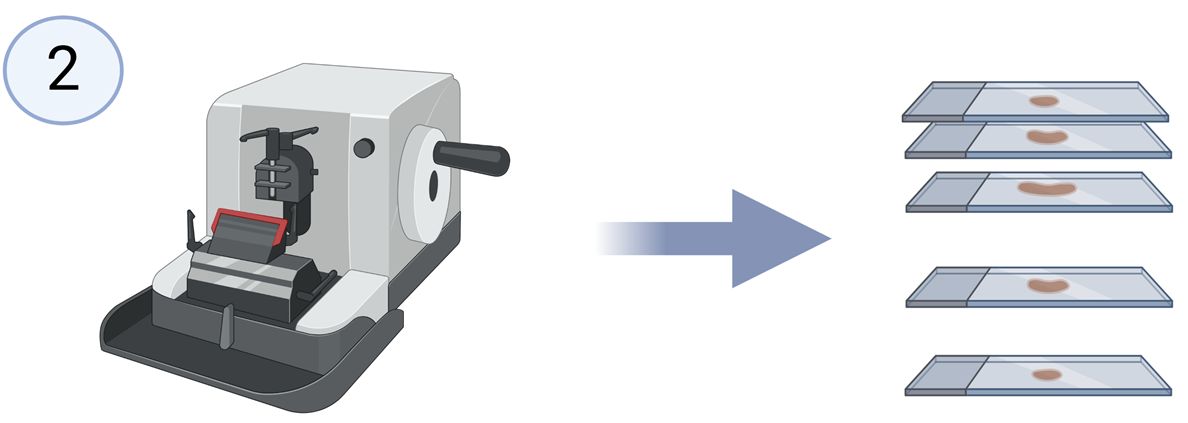
**

1. **Deparaffinize the FFPE slides using a standard procedure (eg. 3x5 min Xylene/ 2x5 min 100% EtOH/ 2x5 min 95% EtOH/ 2x5 min 70% EtOH/ 2x5 min 50% EtOH) and place the slides in PBS (2x5 min).**
2. **Place the slides in a warm (37°C) antigen retrieval solution (0.05% Trypsin, 0.1% CaCl in ddH_2_O; pH 7.8) for 45 minutes.**

**
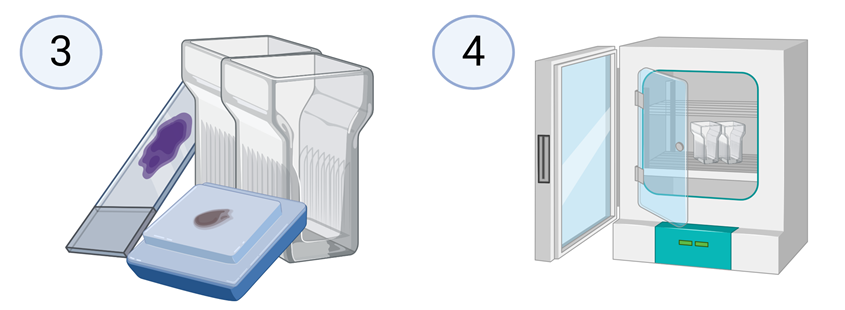

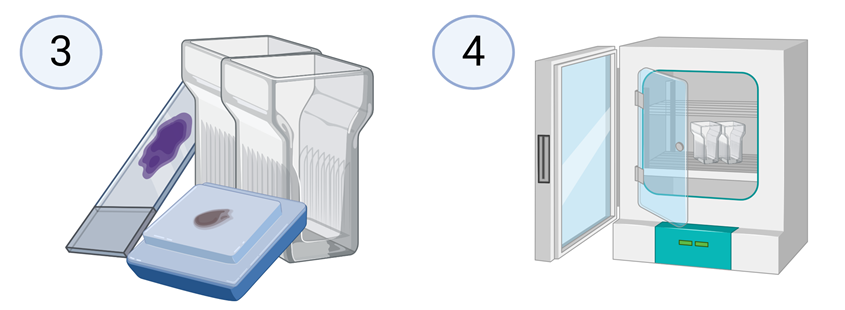
**

1. **Place the slides in PBS (2x5 min) at room temperature to acclimate and prepare the heat-facilitated passive diffusion slice printing setup. The beaker should provide approximately the same pressure as in the protocol for cryosections (31.384 mmHg).**

**
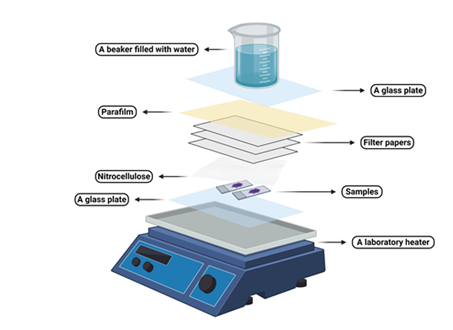
**

1. **Turn on the heater and press the parafilm towards the lower glass plate to ensure optimal isolation and humidity during the transfer protocol.**
2. **The transfer protocol should be modified by the end user; in our case the optimal transfer temperature for rat brain slices was 60°C and the optimal time was determined to be 8 hours.**

**
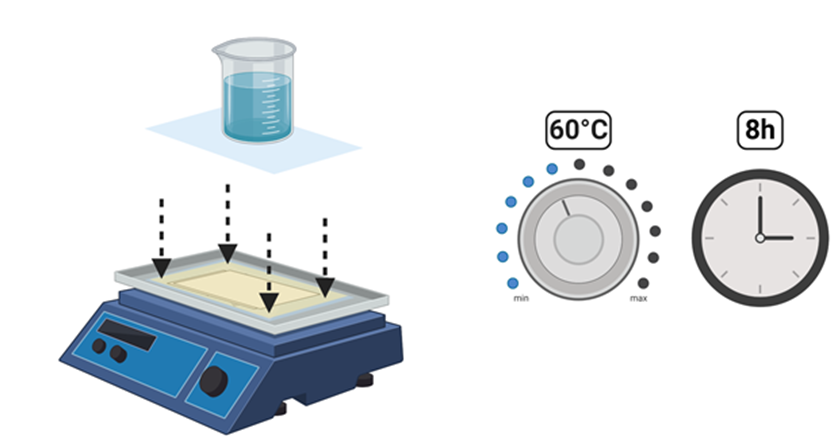
**

1. **After the heat-facilitated passive diffusion slice printing protocol is over, the beaker, upper glass plate, parafilm, and 3 filter papers should be removed, and the membrane should be wetted with PBS, carefully removed with tweezers, and left to dry.**
2. **Once dry, pick up the nitrocellulose membrane with tweezers and place it in the KMnO_4_ working solution for 30 seconds.**
3. **Remove the nitrocellulose membrane from the KMnO_4_ working solution with tweezers and place it under running ddH_2_O to terminate the reaction and increase the contrast.**

**
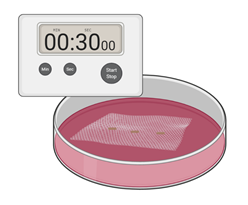

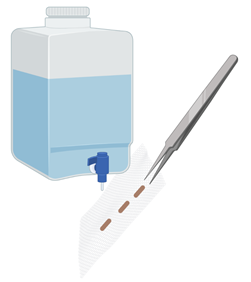
**

1. **Leave the nitrocellulose membrane to dry out and digitalize the membrane (with a cellphone, camera, or an office scanner).**
2. **Import the photo into Fiji (Fiji is just ImageJ) software.**

**
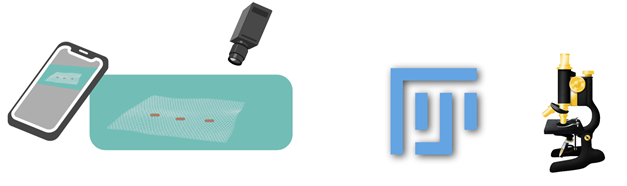
**

1. **Prepare the image for intensity analysis by 8-bit (Image>Type>8-bit) processing and appropriate inversion (Edit>Invert; “CTRL+shift+I”).**

**
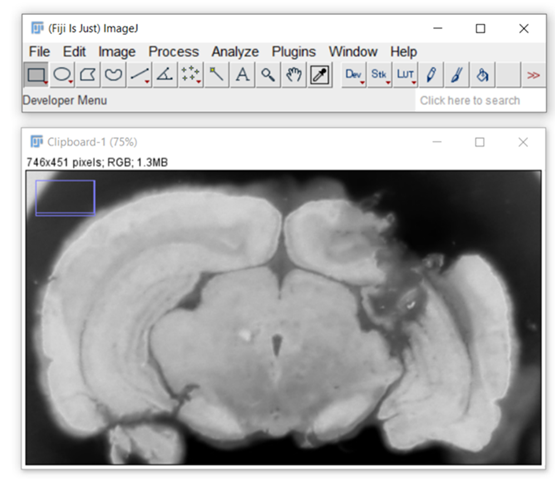
**

1. **Select area of interest using the selection tool and measure pixel intensities by using the Fiji “Histogram” function (Analyze>Histogram; “CTRL+H”).**

**
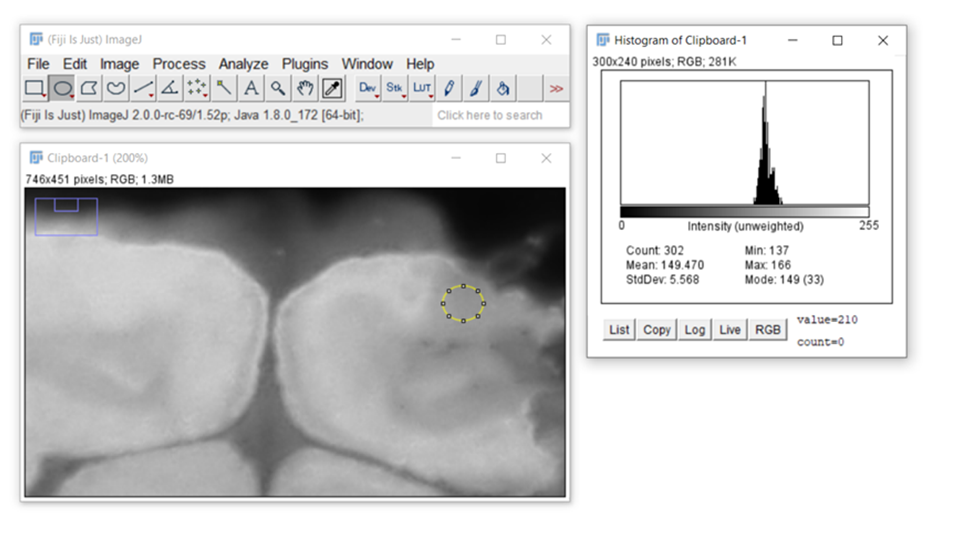
**

1. **Use the “List” option to export values from Fiji for further analysis.**

**
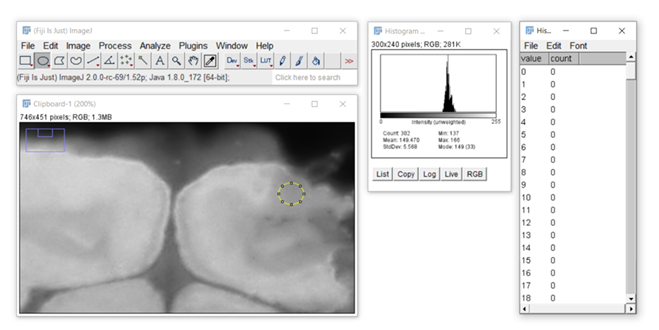
**

1. **An example of pixel intensity comparisons from the ipsilateral side following microdialysis probe-induced damage and the contralateral side of the brain used as a reference. A comparative analysis of 18 brain areas analyzed by density plot comparison done in the R software environment for statistical computing is further explained in the Main text.**

**
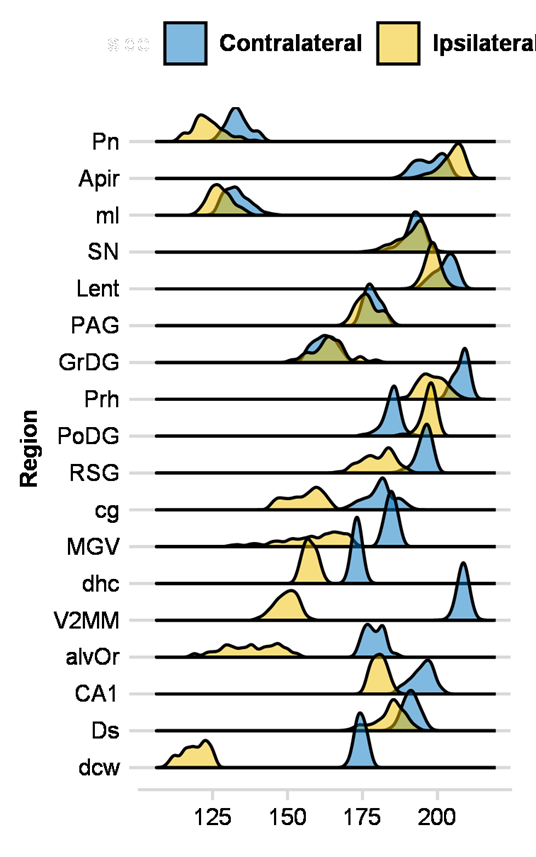
**

# Supplementary material 2: Nitrocellulose redox permanganometry (NRP) linearity, precision, and accuracy validation tests

In order to test the linearity, accuracy, and precision of nitrocellulose redox permanganometry (NRP), we prepared 10 stock solutions of a standard reductive agent, sodium thiosulfate (Na_2_S_2_O_3_), often used for validation of the total antioxidant capacity method. Ten nominal concentrations between 0.1M – 0.01M were prepared independently by weighing out an appropriate amount of Na_2_S_2_O_3_ and dissolving them in double-distilled water (ddH_2_O) with predetermined purity of 0.055 μS/cm. Samples were placed at 4°C until everything was ready for NRP.

The European Medicines Agency Bioanalytical Method Validation Guidelines (EMA-BMVG)[^1^](https://paperpile.com/c/1nYmMD/JWy6) suggest a minimum of 6 samples to be used for the generation of the calibration curve (CC). Here, we used 10 nominal concentrations. Moreover, EMA-BMVG suggests each calibration standard can be analyzed in replicates. Here, 5 technical replicates per concentration were used. It is suggested that a relationship between analyte concentration and the response of the instrument should be reported, and considering that our results had a good linear fit, we reported a standard linear regression equation (y=2100+1.4e6x; R^2^=0.99; p=1.3e-49) (**Main text** **Fig 1B**).

Accuracy was obtained for all ten nominal concentration points following the within-run accuracy determination principles. EMA-BMVG suggest a minimum of 5 samples per level at a minimum of 4 concentration levels which are covering the CC range by the following principle: quality control (QC) samples have to cover the whole concentration curve and include samples reflecting low concentrations (low QC; below 30% of the CC range), medium concentrations (medium QC; around 30-50% of the CC range) and high concentrations (high QC; above 75% of the CC range). Here, we tested within-run accuracy with 5 samples per level at 10 concentration points. More precisely, EMA-BMVG suggests low QC be tested at approximately 3 times the concentration of the lower limit of quantification (LLOQ), the lowest concentration value of analyte in a sample which can be quantified reliably. However, as most of the (biological) samples preliminarily tested fell onto the concentration curve generated by analysis of 0.01M - 0.1M Na_2_S_2_O_3_, we didn’t formally extend our concentration curve looking for the true potential LLOQ concentration point and following the EMA-BMVG principles, our LLOQ point for the within-run Na_2_S_2_O_3_ validation run probably could have been shifted towards much lower values. For example, for within-run accuracy, a 20% deviation from the nominal values is considered acceptable for the LLOQ point. In our case, 0.01 Na_2_S_2_O_3_ taken as LLOQ deviated from nominal values only by 9.47% (**Main text Fig 1B**). Within-run accuracy principles suggest mean concentrations to be within 15% of the nominal values for the low, medium, and high QC, and within 20% for the LLOQ. Our Na_2_S_2_O_3_ QC samples all had a coefficient of variation (CV) below 10%, and all except 0.01M Na_2_S_2_O_3_ had CV below 4% (**Table S2-1**). As Na_2_S_2_O_3_ validation run, CC was calculated from the average integrated density of every QC data point, only CV was used as a validation parameter because mean group values didn’t deviate from the CC values.

As explained in the main text, the whole validation protocol was repeated with another standard reductive agent, ascorbic acid (AA), to ensure that the test truly measured reductive capacity rather than some other property of Na_2_S_2_O_3_. Here, we also decided to test a range of 10 nominal concentrations between 0.1M – 0.01M, although we expected different absolute reductive values. The reason for this was to simply demonstrate two qualities in the same run - the fact that the test reflects the reductive capacity of the tested samples and to show its robustness. In this run, the results obtained from the AA NRP test also had a good linear fit so a standard linear regression equation was reported (y=2700+5.9e5x; R^2^=0.94; p=6.6e-33) (**Main text Fig 1C**). All validation tests were done following the same principles as described for Na_2_S_2_O_3_ validation. The precision of the test was determined by calculating CV for all 10 samples (**Table S2-2**). As expected, CC for AA was much closer to the LLOQ of the NRP. For example, 0.01 M AA had a CV of 21.27%, 1.27% above the recommended LLOQ range. Consequently, for samples with a reductive capacity equal or lower than 0.01M AA in this experiment, the NRP protocol would have to be adjusted to obtain reliable information.

**Table S2-1.** Raw data used for calculation of the Na_2_S_2_O_3_ coefficient of variation in the NRP validation test

| **Sodium thiosulfate concentration [M]** | **Raw NRP values [arbitrary units]** | **CV [%]** |
| --- | --- | --- |
| **0.1** | **125412** | **3.765271544** |
| **0.1** | **129138** |  |
| **0.1** | **131512** |  |
| **0.1** | **138604** |  |
| **0.1** | **133835** |  |
| **0.09** | **122305** | **0.895188076** |
| **0.09** | **121010** |  |
| **0.09** | **121694** |  |
| **0.09** | **121434** |  |
| **0.09** | **119424** |  |
| **0.08** | **110419** | **2.763652803** |
| **0.08** | **112968** |  |
| **0.08** | **114326** |  |
| **0.08** | **117297** |  |
| **0.08** | **118158** |  |
| **0.07** | **106306** | **2.471816343** |
| **0.07** | **103381** |  |
| **0.07** | **101882** |  |
| **0.07** | **101888** |  |
| **0.07** | **99383** |  |
| **0.06** | **83345** | **2.724647765** |
| **0.06** | **84941** |  |
| **0.06** | **86134** |  |
| **0.06** | **87831** |  |
| **0.06** | **89329** |  |
| **0.05** | **76423** | **1.830637261** |
| **0.05** | **77698** |  |
| **0.05** | **77378** |  |
| **0.05** | **77823** |  |
| **0.05** | **74438** |  |
| **0.04** | **51815** | **2.643992359** |
| **0.04** | **50511** |  |
| **0.04** | **53308** |  |
| **0.04** | **54021** |  |
| **0.04** | **53084** |  |
| **0.03** | **42946** | **3.068341054** |
| **0.03** | **43876** |  |
| **0.03** | **45913** |  |
| **0.03** | **44383** |  |
| **0.03** | **42640** |  |
| **0.03** | **42282** |  |
| **0.02** | **27454** | **3.300032665** |
| **0.02** | **27303** |  |
| **0.02** | **26776** |  |
| **0.02** | **28740** |  |
| **0.02** | **28628** |  |
| **0.02** | **28990** |  |
| **0.01** | **14150** | **9.473625281** |
| **0.01** | **14780** |  |
| **0.01** | **14083** |  |
| **0.01** | **12195** |  |
| **0.01** | **11952** |  |
| **0.01** | **9103** |  |

**Table S2-2.** Raw data used for calculation of the ascorbic acid coefficient of variation in the NRP validation test

| **Ascorbic acid concentration [M]** | **Raw NRP values [arbitrary units]** | **CV [%]** |
| --- | --- | --- |
| **0.1** | **55036** | **2.327915513** |
| **0.1** | **58096** |  |
| **0.1** | **56067** |  |
| **0.1** | **54797** |  |
| **0.1** | **56203** |  |
| **0.09** | **54906** | **2.374952486** |
| **0.09** | **56918** |  |
| **0.09** | **57860** |  |
| **0.09** | **58323** |  |
| **0.09** | **57784** |  |
| **0.08** | **44425** | **3.005992443** |
| **0.08** | **47286** |  |
| **0.08** | **47712** |  |
| **0.08** | **47574** |  |
| **0.08** | **47660** |  |
| **0.07** | **44199** | **3.532410204** |
| **0.07** | **47128** |  |
| **0.07** | **47707** |  |
| **0.07** | **48025** |  |
| **0.07** | **49138** |  |
| **0.07** | **46719** |  |
| **0.06** | **37750** | **2.315678687** |
| **0.06** | **39550** |  |
| **0.06** | **39183** |  |
| **0.06** | **39453** |  |
| **0.06** | **37802** |  |
| **0.05** | **33809** | **6.171647379** |
| **0.05** | **36066** |  |
| **0.05** | **36669** |  |
| **0.05** | **39103** |  |
| **0.05** | **39293** |  |
| **0.04** | **27346** | **4.44893066** |
| **0.04** | **30021** |  |
| **0.04** | **30672** |  |
| **0.04** | **30299** |  |
| **0.04** | **29860** |  |
| **0.03** | **22104** | **7.877937712** |
| **0.03** | **24360** |  |
| **0.03** | **25784** |  |
| **0.03** | **27988** |  |
| **0.03** | **26430** |  |
| **0.03** | **25654** |  |
| **0.02** | **9572** | **12.91127875** |
| **0.02** | **10793** |  |
| **0.02** | **12446** |  |
| **0.02** | **13113** |  |
| **0.02** | **10495** |  |
| **0.01** | **1809** | **21.27127437** |
| **0.01** | **1978** |  |
| **0.01** | **2658** |  |
| **0.01** | **2339** |  |
| **0.01** | **2491** |  |
| **0.01** | **3250** |  |

# Supplementary material 3: Nitrocellulose redox permanganometry (NRP) ascorbic acid heating time and temperature response validation experiments

We further tested the specificity of NRP towards the reductive potential to exclude the possibility that the test is in fact reflecting some property related to the concentration of both Na_2_S_2_O_3_ and ascorbic acid (AA). Usually, methods are validated only by concentration curve linearity and precision analysis; however, we believe that the principle of validation that relies exclusively on the concentration-signal relationship analysis is logically flawed as substance concentration bias is unavoidable. In order to overcome this problem, we designed experiments based on the idea of physical oxidation of isoconcentrated samples. Being a classic antioxidant with good NRP linearity and precision (**Main text** **Fig 1C, Supplement 2**), and known for its thermal sensitivity, we decided to use heat-induced decrement of AA reductive capacity as a proof-of-concept for isoconcentrated substance validation. Three experiments were performed. First, we independently prepared six solutions of 0.05M AA and aliquoted each sample into two Eppendorf tubes. One of the two aliquots was placed at 4°C and the other in a heating block at 70°C. After 4 hours, both aliquots were left to acclimate to room temperature and then 1 µl of each solution was placed on a clean sheet of nitrocellulose membrane. Standard NRP protocol was applied and the results were presented in **Fig S3-1A** and **Main text** **Fig 1D**. As expected, reductive capacity was reduced upon heating (p=0.0022). We then moved on to further test this by analyzing the relationship between NRP signal reduction and AA heating time. We prepared 6 replicates of AA samples with three aliquots and placed them either at 4°C for 6.5 hours, at 4°C for 2.5 hours followed by 4 hours at 70°C, or at 70°C for 6.5 hours. Afterward, all samples were left to acclimate to room temperature and then 1 µl of each solution was placed on a clean sheet of nitrocellulose membrane and standard NRP protocol was conducted. The time response of AA oxidation is presented in **Fig S3-1B**. At the temperature of 70°C, most of the AA oxidation occurred in the first 4 hours. Prolongation of AA heating for 2.5 hours provided a small additional decrement of AA reductive potential. In addition, we performed a temperature response experiment to see whether heating at higher temperatures would exert a greater decrement of AA reductive capacity. Here, we independently prepared duplicate samples of 0.1M AA with 4 aliquots and placed the samples for 4 hours at either 4°C, 50°C, 70°C or 95°C. Afterward, all samples were left to acclimate to room temperature and then 1 µl of each solution was placed on a clean sheet of nitrocellulose membrane for NRP. The results are presented in **Fig S3-1C**. Higher temperature resulted in a greater decrease of NRP values. More specifically, 4 hours at 50°C in this experiment reduced AA NRP by approximately 25%, 4 hours at 70°C by 48%, and 4 hours at 95°C by 63%. In conclusion, time and temperature responses of isoconcentrated AA reductive capacity suggest that the results obtained by NRP reflect antioxidant capacity rather than some other concentration-dependent variable.


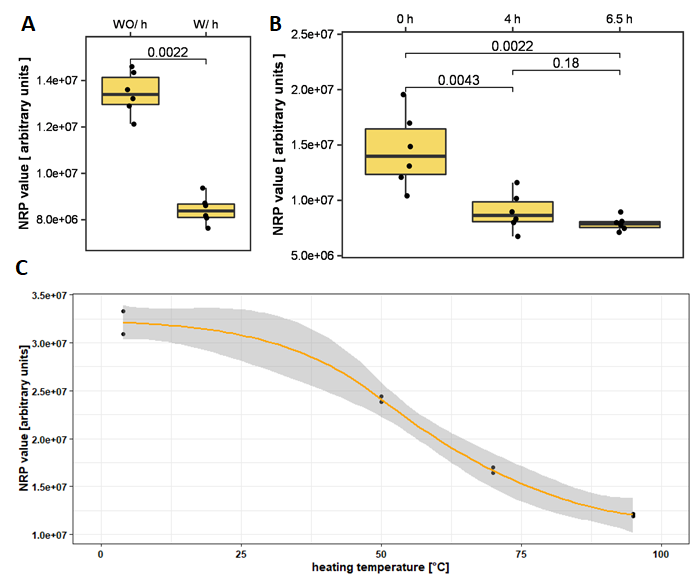


Fig S3-1. Nitrocellulose redox permanganometry (NRP) ascorbic acid heating time and temperature response validation experiments. A) Six independent replicates of 0.05M ascorbic acid (AA) in duplicate aliquots were heated at either 70°C for 4 hours or left at 4°C. After samples were acclimated to room temperature, 1 µl of each solution was placed on a clean sheet of nitrocellulose membrane and analyzed by a standard NRP protocol. B) Six replicates of AA samples with three aliquots were placed at either 4°C for 6.5 hours, at 4°C for 2.5 hours followed by 4 hours at 70°C, or at 70°C for 6.5 hours. After heating, the samples were acclimated to room temperature and analyzed by NRP. C) Independently prepared duplicate samples of 0.1M AA with 4 aliquots were placed for 4 hours at either 4°C, 50°C, 70°C or 95°C. Samples were acclimated to room temperature and analyzed by NRP. NRP-nitrocellulose redox permanganometry; WO/ h-without heating; W/ h-with heating.

# Supplementary material 4: Nitrocellulose redox permanganometry (NRP) membrane stability analysis

Nitrocellulose redox permanganometry is a rapid and simple method for the determination of reductive capacity by quantification of the MnO_2_ precipitate on the nitrocellulose membrane after reducing KMnO_4_ by a nitrocellulose-prefixed sample. After the NRP protocol, the membrane is left to dry. Once sufficiently dry, the NRP membrane is digitalized. Although we suggest that the membrane is digitalized in the first 24 hours, here, we wanted to examine whether the MnO_2_ precipitate is stable and analyzable even after a prolonged delay. Two NRP membranes were digitalized and analyzed two times with a time delay of 9 months. The same HistoNRP membrane with 4 rat brainstem regions analyzed at baseline and after a 9-month delay is presented in **Fig S4-1A**. Although the NRP signal is still conserved even after 9 months, membrane analysis reveals significant fading and signal equalization. An example of the linear sampling pixel intensity profile plot of the approximately same region is visualized in **Fig S4-1B** with the potential source of problematic ratiometric quantification evident for pixels number 15 and 10 in the 9-month delay analysis with ratiometric values for the corresponding baseline pixels being 3.2 and 2.6 respectively. A membrane with six hippocampal homogenates analyzed with standard NRP is presented in **Fig S4-1C** showing both the baseline signal and the same membrane analyzed after 9 months. A significant fading effect was observed. However, in contrast to HistoNRP, time delay in standard NRP resulted in more pronounced differences in contrast to the equalization effect. A possible explanation is fading-induced subtraction of the background signal evident on the baseline membrane (**Fig S4-1C**). Integrated density-based quantification of the membrane is presented in **Fig S4-1D** with corresponding ratiometric values (baseline integrated density over time delay integrated density) for individual samples depicted as data point labels next to baseline values. In conclusion, even though meaningful NRP data can be obtained after an enormous time delay (of at least 9 months), we strongly suggest the analysis be done right after the NRP protocol (in the first 24 hours after the analysis). Time-delay-induced background correction evident in **Fig S4-1C** should be corrected by a longer washing period, shorter incubation in KMnO_4_, or using standard rolling ball background subtraction in Fiji (NIH, USA) [Process>Subtract Background].

Given its practical importance it should be noted that we decided to implement the analysis of NRP membranes in the first 24 hours as an internal standard and not because this is the time-point after which the signal wears off and cannot be analyzed. The 24-hour cut-off time is arbitrary and no differences have been observed at least in the first 10 days following NRP protocol. The most important acute changes of the membrane after once the NRP protocol is done are associated with drying as the MnO_2_ signal becomes slightly less pronounced once the membrane dries off completely. However, once the nitrocellulose membrane is dry, the signal is stable for days and weeks (with no apparent changes). The main reason we reccomend the anaysis to be done in the first 24 hours is to avoid excessive time delay (months), and rough handling of the membrane (e.g. excessive touching of the MnO_2_ precipitate) that may affect the obtained signal.


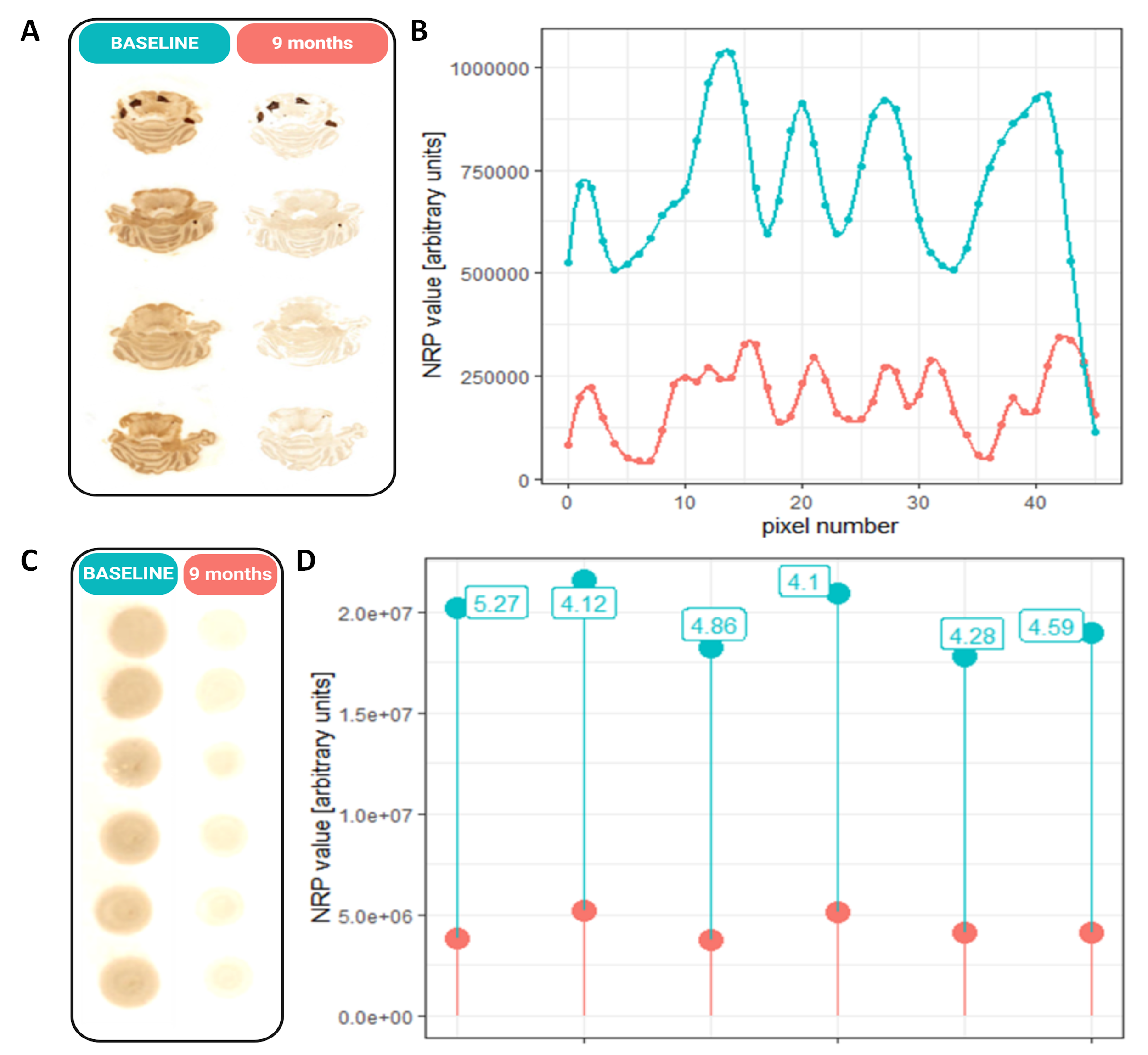


**Fig S4-1. Nitrocellulose redox permanganometry (NRP) membrane stability analysis. A)** HistoNRP membrane with 4 rat brainstem and cerebellum samples digitalized right after the NRP procedure (left) and after a 9-month delay (right). **B)** An example of linear sampling pixel intensity profile plot of the approximately same region right after the NRP procedure (green) and after a 9-month delay (red). **C)** A membrane with six hippocampal homogenates analyzed with a standard NRP right after the NRP procedure (left) and after a 9-month delay (right). **D)** Integrated density-based quantification of the membrane from Fig 1C with baseline values presented in green and values after a 9-month time delay presented in red. Corresponding ratiometric values (baseline integrated density over time delay integrated density) for individual samples are depicted as data point labels next to baseline values.

# Supplementary material 5: Comparison of Nitrocellulose redox permanganometry (NRP) digitalization techniques

Digitalization of the NRP membrane is a prerequisite for reductive capacity assessment based on the quantification of the MnO_2_ precipitate. In the NRP protocol, the precipitate is quantified digitally using image analysis; however, the membrane first has to be transferred into a digital form. As with other digitalization techniques (eg. western blot membranes), it is important that the digital image reflects the true objective state of the membrane. For this reason, we analyzed the linearity of different digitalization techniques knowing all methods will inevitably suffer from some inherent limitations. For example, a simple office scanner was used for most of the experiments, having in mind that most desktop scanners have both a light source and a sensor on the same side of the scanned object, such as paper or in this case, the nitrocellulose membrane, and a reflecting surface on the other. For this reason, the classic principles of the Beer-Lambert law cannot be assumed as the light from the source will pass twice through the membrane and the photoresistor light entrance angle will vary. Nevertheless, we wanted to make NRP as user-friendly as possible, and as precise as possible so various simple digitalization techniques were tested and the best ones were further studied and validated. Four different simple and widely available digitalization techniques are presented in **Fig S5-1.**, and linear regression of the obtained values for a single biological sample diluted in different concentrations. The biological sample used in the experiment was a single hippocampal homogenate of a control animal with a protein concentration of 15.61 μg/μl, as determined by the Lowry assay [^2^](https://paperpile.com/c/iut3IQ/cp7A). Raw images of NRP membranes digitalized with different instruments and corresponding density plots obtained by the Gel Analyzer plugin are shown in **Fig S5-1A-D.** An NRP membrane digitalized by RICOH SP 4410SF desktop office scanner is shown in **Fig S5-1A**, the same membrane digitalized by a Samsung Galaxy S8 cell phone camera with a flash option turned on is presented in **Fig S5-1B,** or with a flash, option turned off in **Fig S5-1C**. Additionally, the membrane was digitalized with a MicroChemi (DNR Bio-Imaging Systems, Israel) camera at -56°C, standardly used for visualization of chemiluminescence with illumination turned on (**Fig S5-1D**). Linear regression with corresponding equations, Pearson correlation coefficients, and p values is shown in **Fig S5-1E**. Based on validation experiments for different digitalization techniques, the office scanner digitalization was chosen as the best method, although Beer-Lambert principles cannot be directly assumed, the method is inherently defined by the enclosed digitalization protocol, important for the maximal reduction of error induced by environmental illumination variability, results are rapidly available, and the method shows good linearity. Results obtained with a Samsung Galaxy S8 cell phone were also very reliable, and even slightly more sensitive in comparison to the scanner. However, considering potential illumination variability differences, we decided to standardly rely on the office scanner for the digitalization of our NRP membranes.

**
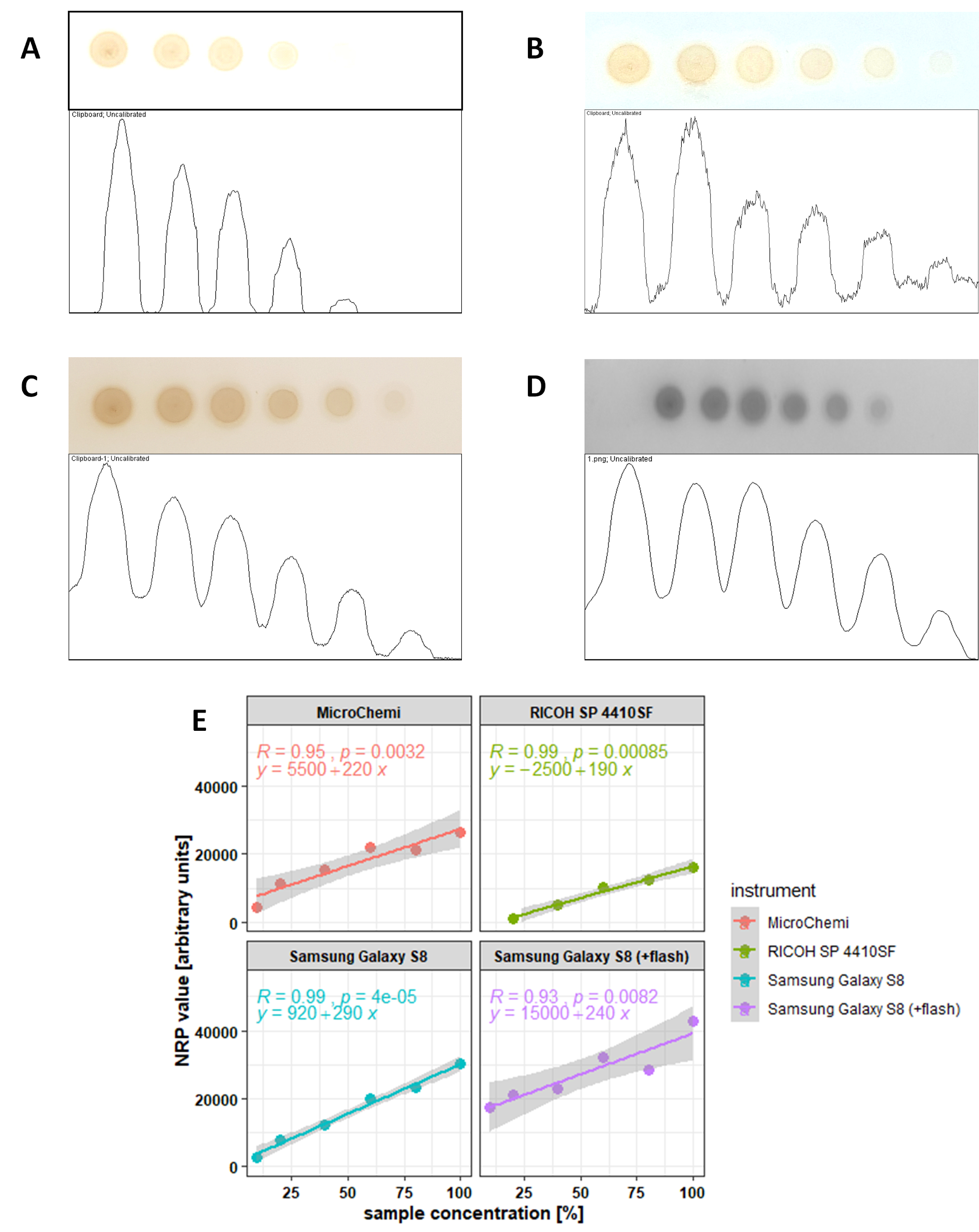
**

**Fig S5-1.** Linearity comparison for digitalization with different instruments. A single NRP membrane was obtained by analysis of different dilutions of a single hippocampal homogenate from the control rat (1,0.8, 0.6, 0.4, 0.2, 0.1). **A)** An NRP membrane and corresponding density plot digitalized by the RICOH SP 4410SF desktop office scanner. **B)** An NRP membrane and corresponding density plot digitalized by a Samsung Galaxy S8 cell phone camera with a flash option turned on. **C)** An NRP membrane and corresponding density plot digitalized by a Samsung Galaxy S8 cell phone camera with the flash option turned off. **D)** An NRP membrane and corresponding density plot digitalized by the MicroChemi (DNR Bio-Imaging Systems, Israel) camera at -56°C, standardly used for visualization of chemiluminescence with illumination turned on. **E)** Linear regressions with corresponding equations, Pearson correlation coefficients, and p values for data obtained following digitalization with different instruments. All density plots were obtained by the Gel Analyzer plugin for Fiji (NIH, USA) and data were extracted by measuring the area under the curve.

# Supplementary material 6: An explanation of the computational analysis of Nitrocellulose redox permanganometry (NRP)

To obtain accurate results of sample reductive capacity, adequate digitalization and analysis of the membrane are indispensable. This document provides a step-by-step protocol on how to analyze NRP membranes to ensure proper implementation of NRP into research practice. A single NRP membrane digitalized by RICOH SP 4410SF desktop office scanner as described in **Supplement 6** was used here for demonstrative purposes. Although different software solutions can be used for signal quantification, we recommend Fiji (Fiji Is Just ImageJ), a version of the popular ImageJ software (NIH, USA) with pre-installed plugins. Precise quantification of the NRP signal can be simply done by using the Fiji Gel Analyzer plugin and following the same protocol as recommended for the dot blot analysis. In the following protocol, the steps are shown in green, and the corresponding ImageJ macro code is shown in red.

1. Import the membrane image into Fiji.

[File>Open…]
[open("D:/Users/jan.homolak/Desktop/SampleNRP.png");]

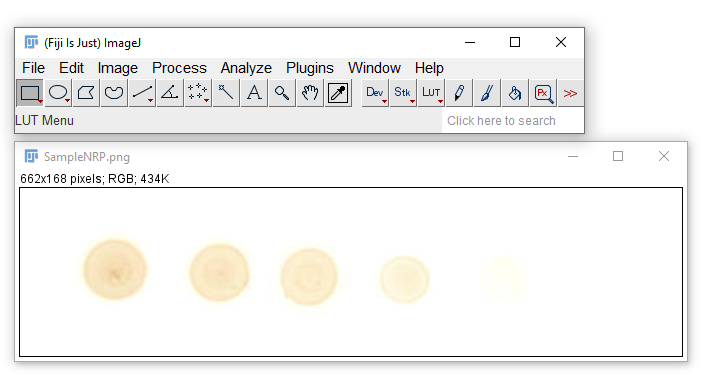


1. Use the rectangle tool to select samples

[Rectangle]
[//setTool("rectangle");
makeRectangle(9, 19, 602, 131);]


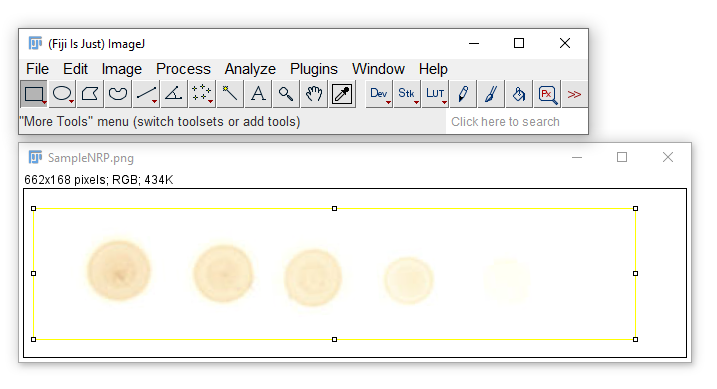


1. Use the Gel Analyzer “Select First Lane” option

[Analyze>Gels>Select First Lane//OR//CTRL+1]

[run("Select First Lane");]


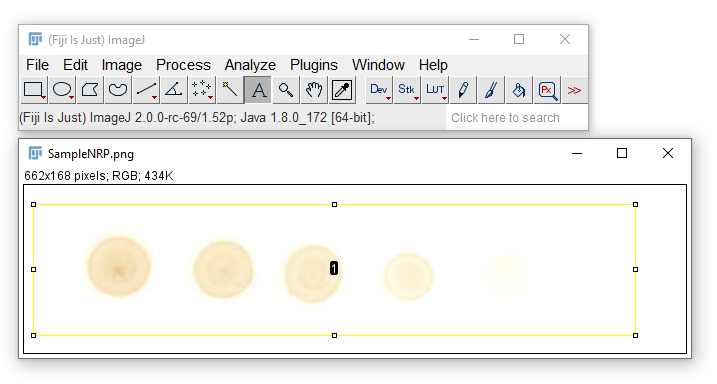


1. Use the Gel Analyzer “Plot Lanes” option

[Analyze>Gels>Plot Lanes//OR//CTRL+3]

[//setTool("line");
run("Plot Lanes");]


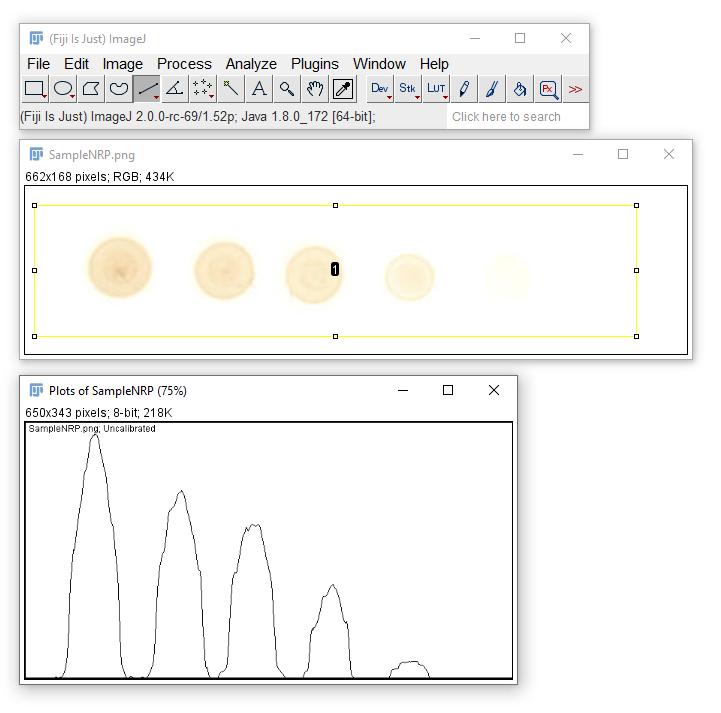


1. Use the Wand tool to select density plots of the corresponding samples and calculate the area under the curve

[Wand tool]

[//setTool("wand");
doWand(94, 189);

doWand(209, 253);

doWand(298, 220);

doWand(420, 282);

doWand(518, 328);]


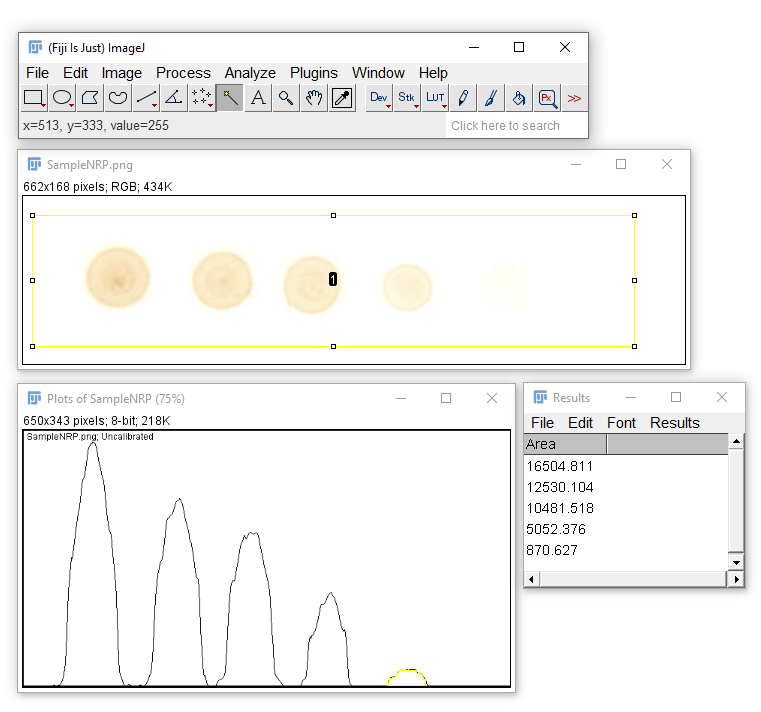


1. Select the values in the Results window and save the results for subsequent analysis and visualization. Alternatively, you can select the results with the pointer and copy and paste them into an excel sheet
   [Results:File>Save As...//OR//CTRL+S]

[saveAs("Results", "D:/Users/jan.homolak/Desktop/SampleNRPresults.csv");]


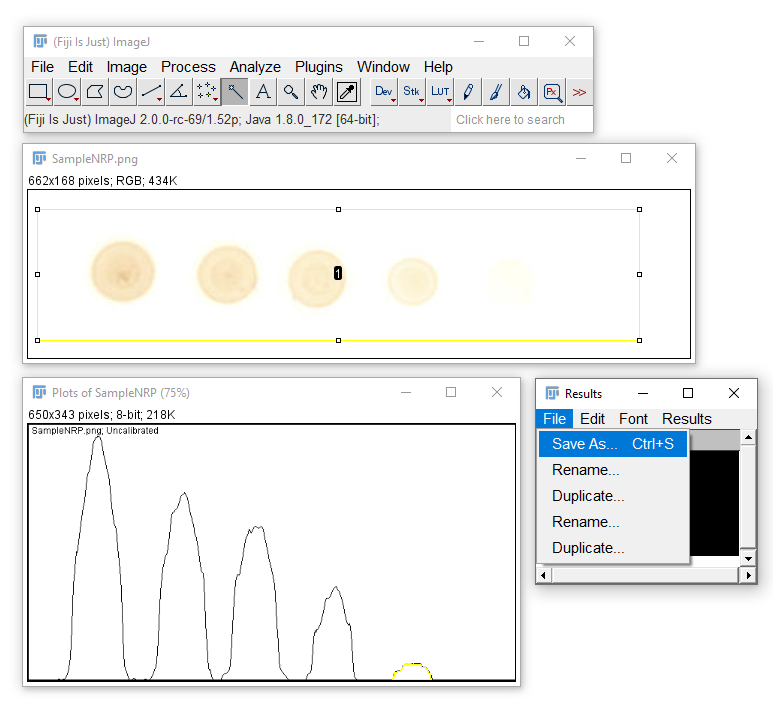


1. Alternatively, you can directly plot the results inside Fiji.
   [Results:Results>Plot…]
   [Plot.create("Plot of Results", "x", "Area");

Plot.add("Connected Circles", Table.getColumn("Area", "Results"));

Plot.setStyle(0, "red,#a0a0ff,2.0,Connected Circles");]


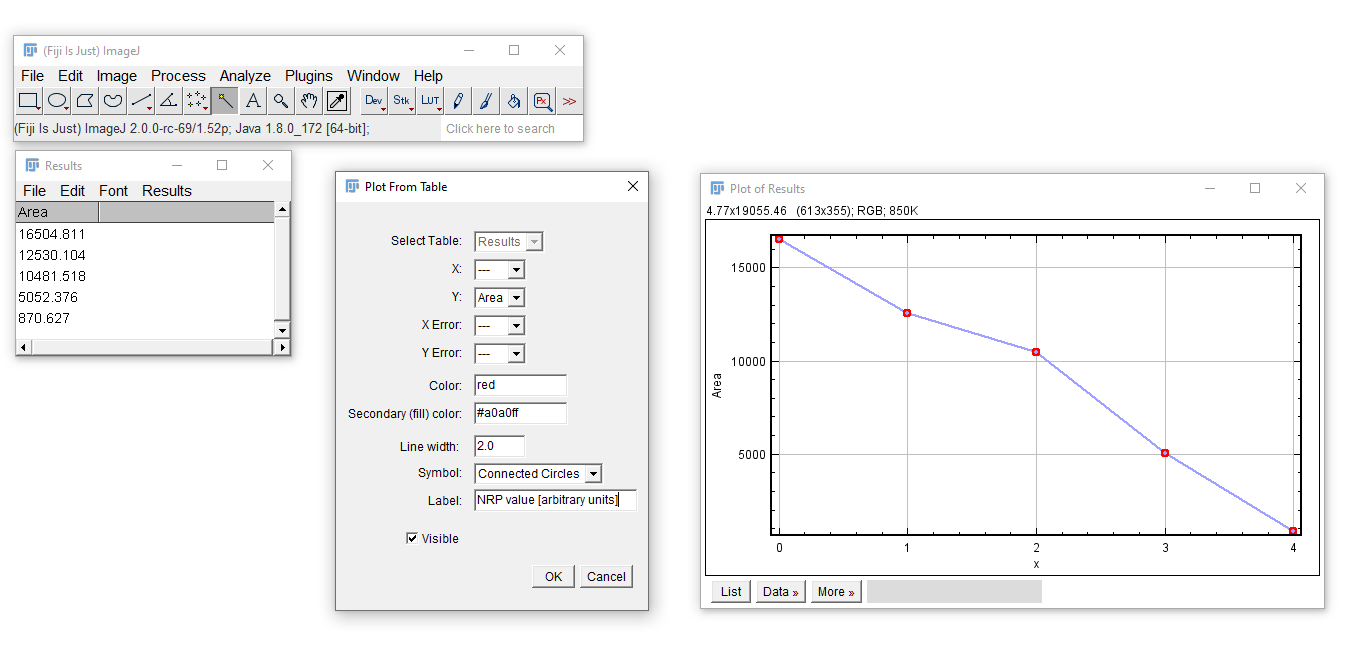


1. Further analysis can also be done directly on the obtained results. An example of regression analysis is shown.

[Plot of Results:Data>>Add fit]

[Plot.setStyle(2, "red,#a0a0ff,2.0,Line");]


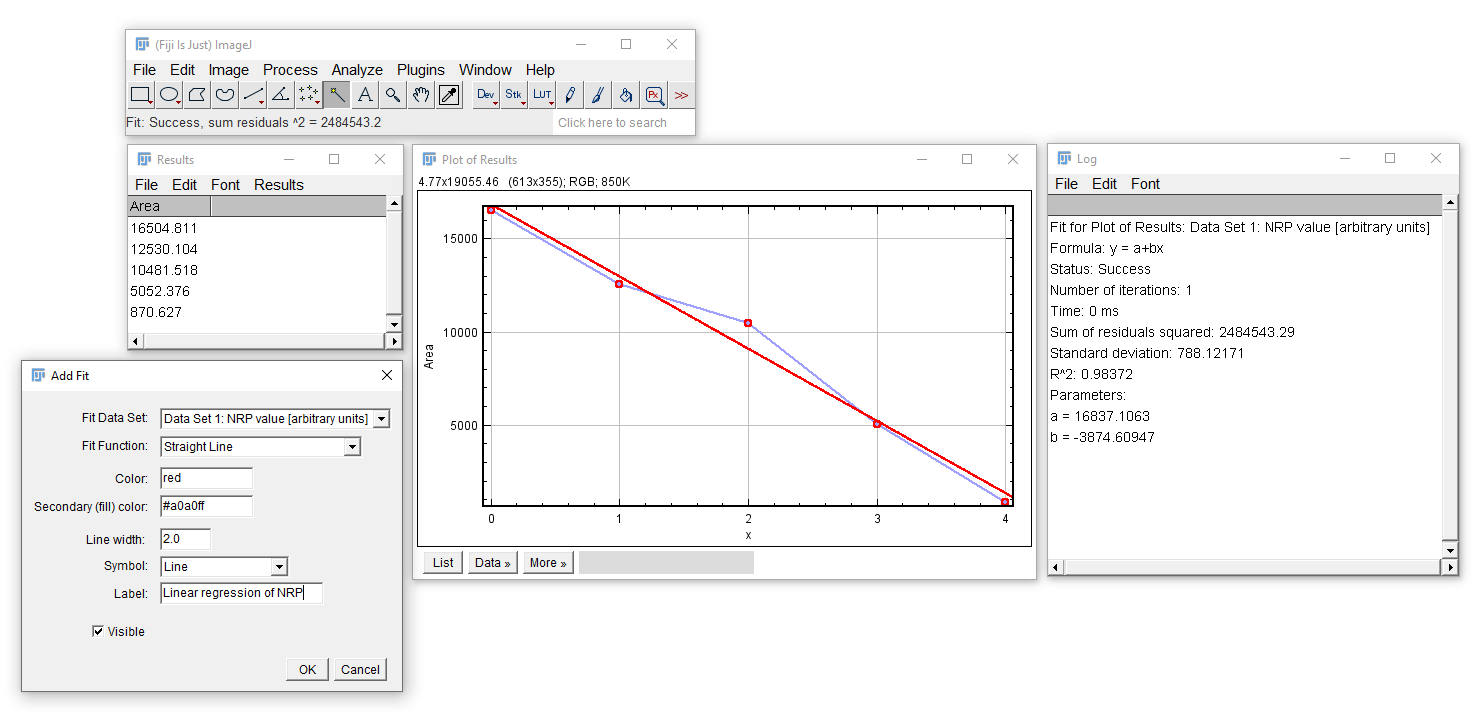


1. Additional visual 3D representation of the NRP signal can also be done in Fiji by using the 3D Surface Plot Analysis option

[File>Open…
Analyze>3D Surface Plot]

[run("3D Surface Plot");]


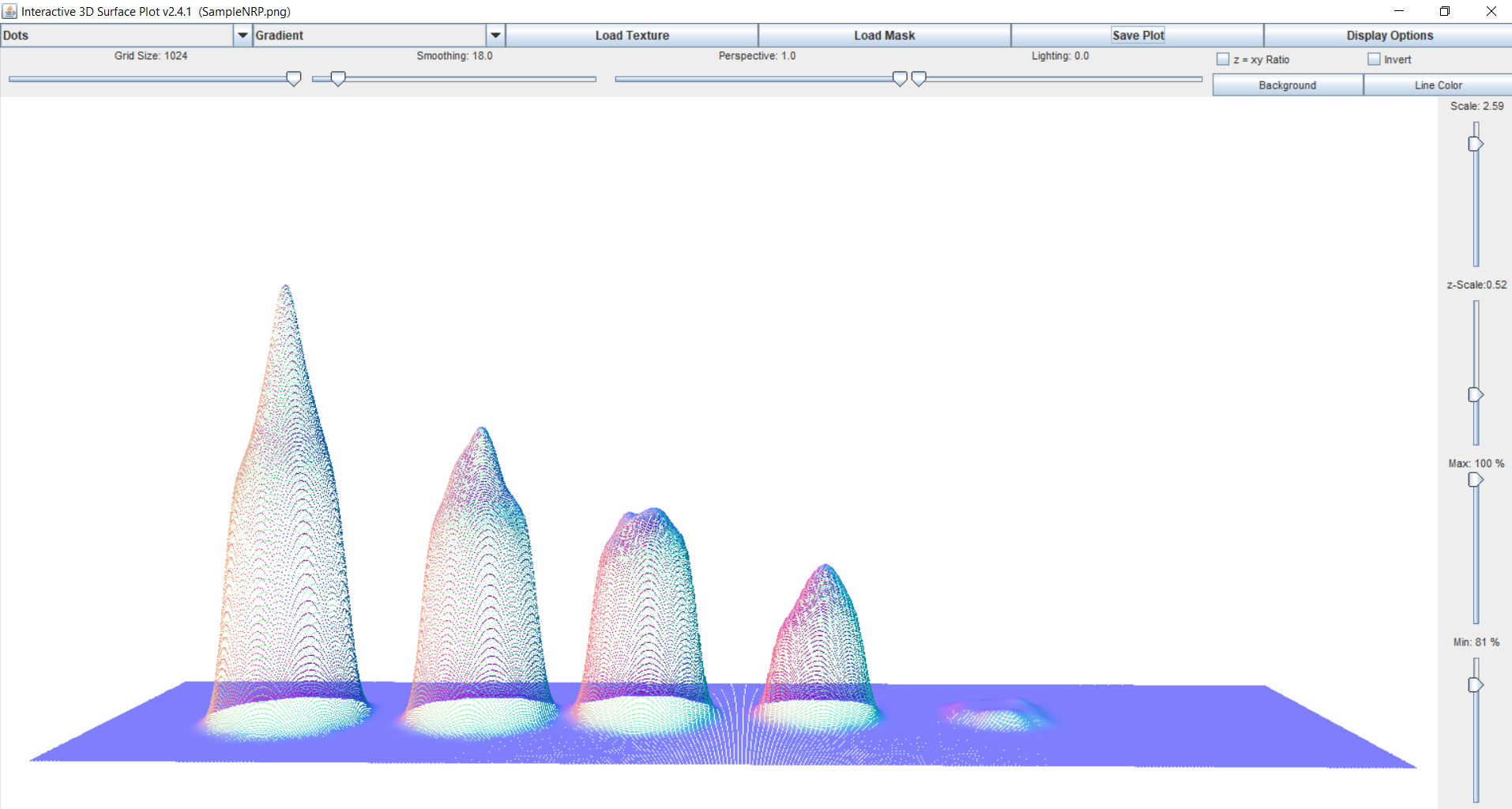


# Supplementary material 7: Additional replications of ORP-NRP experiments from Fig 1E and Fig 1F

**
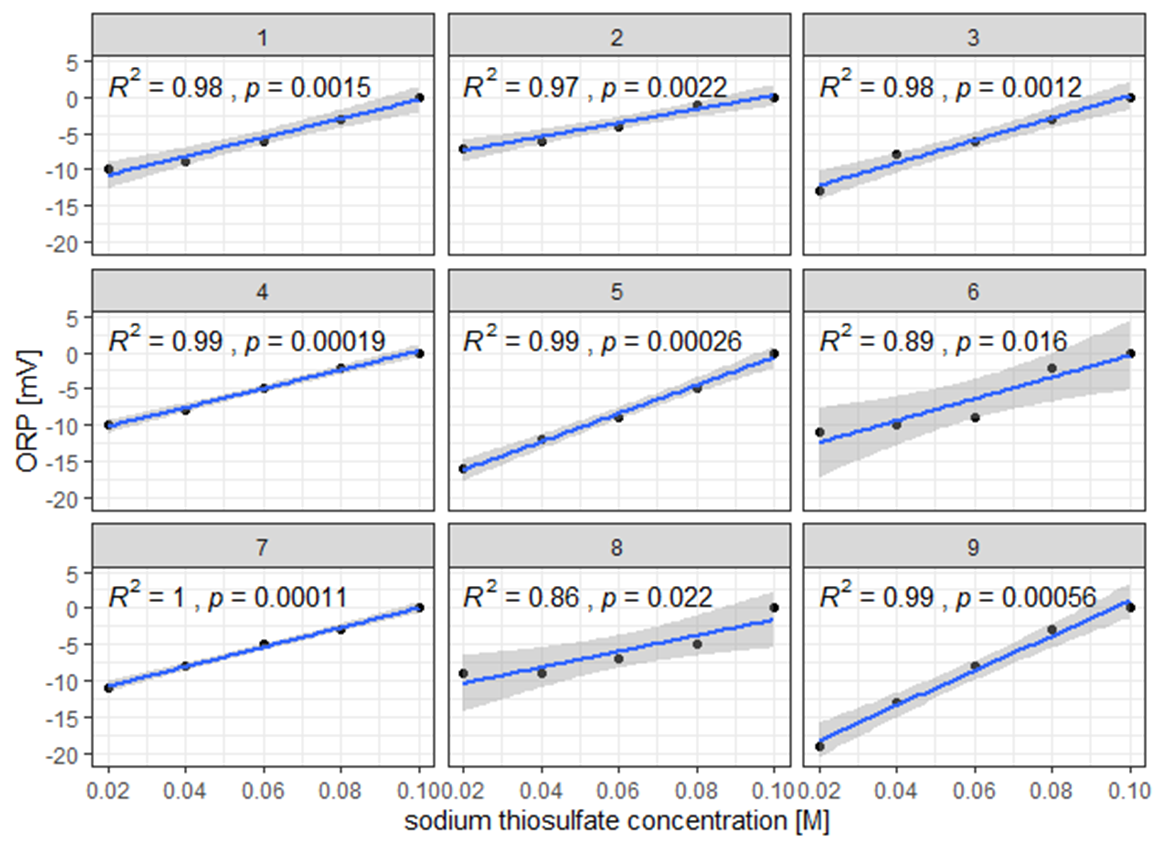

Fig S7-1.** Additional replications of the experiment presented in Fig 1E in the Main text.

**
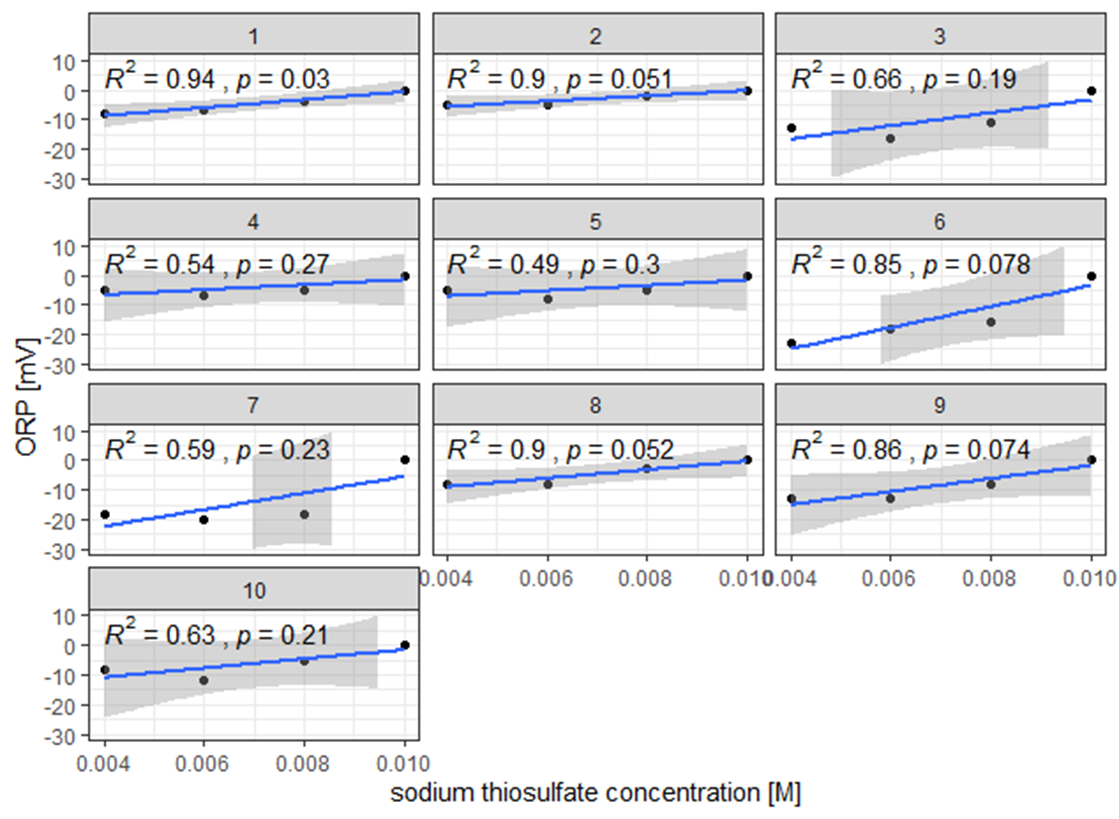
**

**Fig S7-2.** Additional replications of the experiment presented in Fig 1F in the Main text.

# Supplementary material 8: A detailed explanation of the origin of animal tissue used in the Nitrocellulose redox permanganometry (NRP) proof of concept experiments

In short, animal tissue from 3 different animal experiments was used in the NRP proof of concept studies. A single hippocampal homogenate from a single control animal was used for the analysis of exogenous redox system manipulation of the biological specimen illustrated in the main text in **Fig 1F**. The sample we used was obtained from **Experiment 1**. The sample was used in **Supplement 6** (A comparison of NRP membrane digitalization techniques) and in **Supplement 10** (Protein content correction analysis). Plasma and hippocampal homogenates obtained from 6 control rats and 6 rats treated intracerebroventricularly with streptozotocin (STZ-icv) were used for proof of concept of biological specimen NRP analysis presented in **Fig 2A** and **Fig 2C**. Plasma and hippocampal homogenates from 6 wild-type mice and 6 transgenic tg2576 animals was used for additional proof of concept of NRP analysis of biological samples. These samples were obtained in **Experiment 2**. Finally, the tissue used for demonstration of HistoNRP shown in **Fig 2F-J**, as well as tissue used for NRP membrane stability analysis shown in **Supplement 4** was harvested in **Experiment 3**. Here, 6 hippocampal homogenates of untreated rats were used (**Supplement 4**), as well as tissue sections of the brain stem region from 4 animals (**Supplement 4**) and 1 coronal section of the rat brain-damaged unilaterally with a microdialysis probe (**Main text Fig 2F-J**).

Experiment 1:

The experiment aimed to assess acute effects of oral and intraperitoneal galactose load in a rat model of sporadic Alzheimer’s disease. Forty male Wistar rats, bred and kept in-house (Department of Pharmacology, University of Zagreb School of Medicine) were used for the experiment. The animals were kept in an animal facility with stable conditions (constant temperature and humidity – 22-24⁰C and 40-60%, and 12-h light/12-h dark cycle) in standard cages with wood-chip bedding and food and water ad libitum; with three animals placed per cage. At the age of three months, the animals underwent intracerebroventricular treatment with either streptozotocin (STZ-icv; 30 animals) or 0.05M citrate buffer, pH 4.5 (CTR; 10 animals). STZ is a beta-cytotoxic substance selective for insulin-secreting/producing cells, which has been abundantly used to induce an insulin-resistant brain state in laboratory animals, mimicking the main hallmarks of sporadic Alzheimer’s disease. STZ was applied intracerebroventricularly to rats in deep anesthesia (ketamine 50 mg/kg / xylazine 5 mg/kg, ip) in a dose of 3 mg/kg divided in two doses (48 hours apart) into the rats’ lateral ventricles (according to the procedure first described by Noble et al. [^3^](https://paperpile.com/c/UfqKth/oEmy) and used by our research team in previous experiments [^4–^](https://paperpile.com/c/UfqKth/rSyq+fPZT+M1pV)^6^, whereas control animals received only the vehicle, citric buffer, in the same manner. One month after STZ-icv treatment, 20 STZ-icv treated rats were divided into 2 groups, with 10 animals per group, and received either a single oral dose of galactose via gastric tube (200 mg/kg dissolved in 1 mL of water, equivalent to a 6% galactose solution) or a single ip dose (200 mg/kg dissolved in 1 mL of saline solution, equivalent to a 6% galactose saline solution), adjusted according to the rats’ body weight. The remaining 10 CTR and 10 STZ animals were left as intact controls. Fifteen minutes after the galactose load (time point chosen based on literature data [^7^](https://paperpile.com/c/UfqKth/jCWY) due to rapid conversion of galactose to glucose in the blood), the animals were anesthetized with a combination of ketamine and xylazine and blood samples were taken from the retro-orbital sinus. Whole blood samples were collected in heparinized tubes, centrifuged at 3600 RPM for 10 minutes, and the resulting supernatants frozen and stored at -80⁰C. Rats were then sacrificed in two ways, pertaining to the purpose of the collected tissue. Four animals per group, while still in deep anesthesia, underwent transcardial fixative perfusion [^8^](https://paperpile.com/c/UfqKth/rr7F), where the tissue is first washed with saline and subsequently fixated with 4% buffered paraformaldehyde. Upon removal of the intestines and brains, the tissue is submerged in a 15% sucrose-buffered paraformaldehyde solution followed by 30% sucrose-phosphate buffer solution immersion and consequent freezing at -80⁰C for long-term storage. The remaining six animals per group underwent cervical dislocation and their intestines and brains were quickly removed and dissected on ice, with the removal of the hippocampi, freezing using liquid nitrogen and storage at -80⁰C. Hippocampal samples were later thawed and ultrasonically homogenized in a lysis buffer containing 50 mM Trizma base (pH 8), 150 mM sodium chloride, 0,5 mM EDTA, 1 mM dithiothreitol, 0,01 M sodium vanadate, 0,5% sodium deoxycholate, 1% NP-40 detergent and 0,1% SDS. The sonicated samples were then centrifuged at 12000 RPM for 10 minutes, and total protein content from the separated supernatants was measured using the Lowry method [^9^](https://paperpile.com/c/UfqKth/Nlvx). Finally, supernatants were stored at -80⁰C until further analysis.

## Experiment 2:

The experiment was devised to examine the potential therapeutic effects of oral galactose treatment in a mouse model of familial Alzheimer’s disease. Twenty adult male B6; SJL-Tg(APPSWE)2576 Kha heterozygous transgenic mice (TG) overexpressing the human amyloid precursor protein (APP), and 20 corresponding wild types (WT) were purchased from Taconic Biosciences Inc. (Hudson NY, USA). From the age of 3 months onwards, mice were individually housed in appropriate cages with wood-chip bedding, which was located in ventilated cabinets to ensure stable temperature (22–24 °C) and humidity (40–60%) conditions. The cabinets were placed in an air-conditioned room with a 12-h light/12-h dark cycle, specialized for housing transgenic animals (Croatian Institute for Brain Research, University of Zagreb School of Medicine). At the age of 10 months, when, according to literature data [^10,^](https://paperpile.com/c/UfqKth/kku8+FYk6)^11^, this model develops cognitive deficits and neurochemical changes, but no plaque accumulation is evident, the animals entered the experiment, and baseline cognitive and metabolic parameters were measured before initiation of galactose therapy. Cognitive function was assessed using the Morris Water Maze (MWM) swimming test for spatial learning [^12^](https://paperpile.com/c/UfqKth/51pi), nesting behavior was evaluated by scoring the quality of the animals’ nest formation from the given material per standard protocol [^1^](https://paperpile.com/c/UfqKth/yWPZ)^3^, glucose metabolism and homeostasis were measured by intraperitoneal glucose tolerance testing (ipGTT) in fasting mice [^1^](https://paperpile.com/c/UfqKth/xApS)^4^ and anxiety-related behavior was assessed by standard elevated plus-maze test (EPM) [^1^](https://paperpile.com/c/UfqKth/TlVH)^5^. After the establishment of baseline values, mice were divided equally into 4 groups based on cognitive results, with 10 animals per group. One TG and one WT group started 2-month oral galactose therapy, whereas the remainder of the animals kept receiving tap water ad libitum. Oral galactose (Sigma Aldrich, USA) was dissolved in tap water at a dose of 200 mg/kg/day; a dose selected based on previous experiments where it successfully prevented/normalized early cognitive decline in an intracerebroventricular-streptozotocin model of sporadic Alzheimer’s disease in rats [^16,1^](https://paperpile.com/c/UfqKth/0tTN+Skoy)^7^. After 2 months of oral galactose treatment, mice underwent MWM, passive avoidance testing (PAT) for aversive memory assessment [^1^](https://paperpile.com/c/UfqKth/A8HY)^8^, nesting-behavior assessment, ipGTT, EPM, open field testing (OF) for locomotor and anxiety-like behavior [^1^](https://paperpile.com/c/UfqKth/kqrz)^9,^ and in vivo brain glucose uptake measurement of 18fluorodeoxyglucose by PET scan (FDG-PET; 4 animals per group). Upon the completion of cognitive and metabolic tests, mice were anesthetized with a combination of ketamine and xylazine and blood samples were taken from the retro-orbital sinus. Whole blood samples were collected in heparinized tubes, centrifuged at 3600 RPM for 10 minutes, and the resulting supernatants frozen and stored at -80⁰C. Mice were then sacrificed in two ways, pertaining to the purpose of the collected tissue. Four animals per group, while still in deep anesthesia, underwent transcardial fixative perfusion [^8^](https://paperpile.com/c/UfqKth/rr7F), where the tissue is first washed with saline and subsequently fixated with 4% buffered paraformaldehyde. Upon removal of the intestines and brains, the tissue is submerged in a 15% sucrose-buffered paraformaldehyde solution followed by 30% sucrose-phosphate buffer solution immersion and consequent freezing at -80⁰C for long-term storage. The remaining six animals per group underwent cervical dislocation and their intestines and brains were quickly removed and dissected on ice, with the removal of the hippocampi, freezing using liquid nitrogen and storage at -80⁰C. Hippocampal samples were later thawed and ultrasonically homogenized in a lysis buffer containing 50 mM Trizma base (pH 8), 150 mM sodium chloride, 0,5 mM EDTA, 1 mM dithiothreitol, 0,01 M sodium vanadate, 0,5% sodium deoxycholate, 1% NP-40 detergent and 0,1% SDS. The sonicated samples were then centrifuged at 12000 RPM for 10 minutes, and total protein content from the separated supernatants was measured using the Lowry method [^9^](https://paperpile.com/c/UfqKth/Nlvx). Finally, supernatants were stored at -80⁰C until further analysis.

## Experiment 3:

The experiment aimed to assess acute metabolic and redox regulatory network time-response (up to 2h) after oral galactose load in rats. Forty male Wistar rats, bred and kept in-house (Department of Pharmacology, University of Zagreb School of Medicine) were used for the experiment. The animals were kept in an animal facility with stable conditions (constant temperature and humidity – 22-24⁰C and 40-60%, and 12-h light/12-h dark cycle) in standard cages with wood-chip bedding and food and water ad libitum; with three animals placed per cage. At the age of 3 months, the animals were randomized into 5 groups as follows: baseline control group (CTR; n=8), 3 galactose treatment groups sacrificed either in 0.5h time point (n=8), 1h time point (n=8), 2h time point (n=8), and an additional group also sacrificed 2 hours after the galactose treatment but with a preinstalled microdialysis probe (n=8). The microdialysis probe was installed 24 hours before the experiment. In short, rats were anesthetized with isoflurane in the Isoflurane Vaporizer (Ugo Basile, Italy) and placed in the stereotaxic apparatus (Stoelting, USA). A microdialysis probe (CMA Microdialysis AB, Sweden) was placed and fixed with GC FujiCEM (GC Corporation, Japan). 50 mg/ml of metamizole was administered for postsurgical analgesia. On the day of the experiment, rats either received no treatment (CTR) or received 200 mg/kg of galactose dissolved in 1 mL of water by orogastric gavage and were sacrificed either 0.5, 1, or 2 hours after the treatment. Animals were anesthetized with a combination of ketamine and xylazine and blood samples were taken from the retro-orbital sinus. Whole blood samples were collected in heparinized tubes, centrifuged at 3600 RPM for 10 minutes, and the resulting supernatants frozen and stored at -80⁰C. Rats were then sacrificed in two ways, pertaining to the purpose of the collected tissue. Four animals per group, while still in deep anesthesia, underwent transcardial fixative perfusion [^8^](https://paperpile.com/c/UfqKth/rr7F), where the tissue is first washed with saline and subsequently fixated with 4% buffered paraformaldehyde. Upon removal of the intestines and brains, the tissue is submerged in a 15% sucrose-buffered paraformaldehyde solution followed by 30% sucrose-phosphate buffer solution immersion and consequent freezing at -80⁰C for long-term storage. The remaining six animals per group underwent cervical dislocation and their intestines and brains were quickly removed and dissected on ice, with the removal of the hippocampi, freezing using liquid nitrogen and storage at -80⁰C. Hippocampal samples were later thawed and ultrasonically homogenized in a lysis buffer containing 50 mM Trizma base (pH 8), 150 mM sodium chloride, 0,5 mM EDTA, 1 mM dithiothreitol, 0,01 M sodium vanadate, 0,5% sodium deoxycholate, 1% NP-40 detergent and 0,1% SDS. The sonicated samples were then centrifuged at 12000 RPM for 10 minutes, and total protein content from the separated supernatants was measured using the Lowry method [^9^](https://paperpile.com/c/UfqKth/Nlvx). Finally, supernatants were stored at -80⁰C until further analysis.

Some of the results from Experiment 1 and Experiment 2 were published in [^16,1^](https://paperpile.com/c/UfqKth/0tTN+Skoy)^7^.

# Supplementary material 9: Nitrocellulose redox permanganometry (NRP) protein concentration-based correction analysis

Biological sample concentration correction is often done by normalization to total protein content. A simple ratiometric correction assumes a linear relationship between measured properties and protein content. However, this assumption is rarely tested directly. In order to make sure ratiometric correction for protein content is a valid approach for normalization of NRP values obtained from biological samples with different protein concentrations we designed an experiment to test linearity between protein concentration and NRP values. In short, a single biological sample (hippocampal homogenate from a single control rat from **Experiment 1**) with a protein concentration of 15.61 μg/μl as determined by folin phenol protein quantification [^9^](https://paperpile.com/c/b7RRw4/iyy7)was diluted at 80, 60, 40, 20 and 10% of its original concentration with ddH_2_O to make a set of samples for linearity comparisons. Samples (1 μl) were pipetted onto the nitrocellulose membrane in duplicates and the membranes were left to dry. Once dry, one membrane was stained with the 0.1% (w/v) Ponceau S in 5% (v/v) acetic acid [^20,2^](https://paperpile.com/c/b7RRw4/bLeY+MHV1)^1^ for additional protein quantification, and standard NRP protocol was used on the other membrane. Both membranes were scanned and analyzed in Fiji with the Gel Analyzer plugin (NIH, USA). Analysis of measured and predicted NRP values following the principle of maximal linearity and corresponding percent deviations are illustrated in **Fig S9-1A.** As evident from the graph even from the protein concentrations equal to 40% of the original concentration, a deviation from maximal linearity was just 12.7% so we concluded a ratiometric approach is justified for protein concentrations deviating from the standard concentration by approximately 50% of the nominal value. Next, we were interested in the source of variation to see whether dispersion increasing with a factor of deviation from the nominal point is inherently related to NRP, or to the principle of nitrocellulose digital quantification analysis so we compared the values obtained with NRP to the values of signal intensity quantification for the same samples analyzed with Ponceau S nitrocellulose protein staining (**Fig S9-1B**). Interestingly, NRP values demonstrated better linearity and less dispersion from the absolute linear principles in comparison with the Ponceau S nitrocellulose protein quantification.


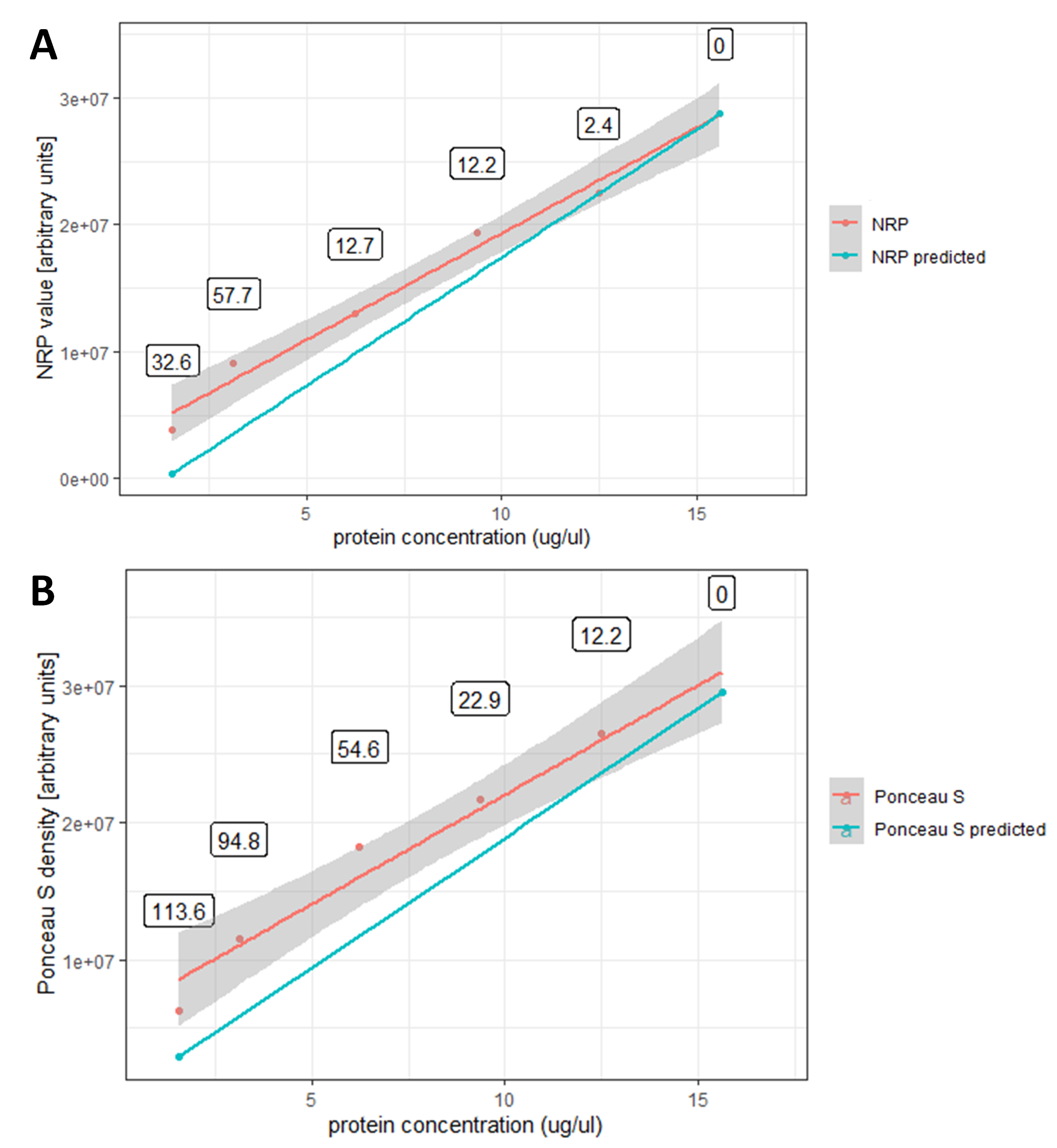


**Fig S9-1.** Comparison of predicted and obtained NRP **(A)** and Ponceau S **(B)** values from serial dilutions of a biological sample.

# Supplementary material 10: HistoNRP adaptation for the analysis of formalin-fixed paraffin-embedded (FFPE) tissue sections

Measurements of spatial reductive capacity of tissue sections using the HistoNRP method largely depend on the success of transfer of the molecular content of tissue onto the nitrocellulose membrane. In order to ensure optimal transfer from the FFPE sections, a standard HistoNRP protocol explained in Supplement 1 has to be adapted. The main difference between HistoNRP for cryosections and FFPE HistoNRP is an additional trypsin-based enzymatic retrieval step, and the introduction of heat-facilitated passive diffusion slice printing step instead of classic passive diffusion slice print blotting used for the transfer of cryosections onto the nitrocellulose membrane. In short, FFPE tissue is first cut on the microtome and tissue sections are mounted onto the histological slides and processed by the standard deparaffinization protocol through Xylene followed by decreasing concentrations of ethanol (EtOH)(3x5 min Xylene/ 2x5 min 100% EtOH/ 2x5 min 95% EtOH/ 2x5 min 70% EtOH/ 2x5 min 50% EtOH). Finally, sections are immersed in phosphate-buffered saline (PBS; pH 7.4) 2 times for 5 minutes. After PBS, the sections are placed in warm (37°C) antigen retrieval solution (0.05% Trypsin, 0.1% CaCl in ddH_2_O; pH 7.8) for 45 minutes. After the retrieval, the sections are placed in PBS for 2x5 min at room temperature (RT) to equilibrate. After equilibration, a glass plate is placed on the laboratory heater and the sections are placed on top of the glass plate and wetted with PBS. A piece of nitrocellulose membrane is placed on top of the tissue sections, and 3 filter papers are placed on the nitrocellulose membrane and wetted with PBS. Finally, a piece of parafilm is placed on top of wet filter papers, and the edges are pressed towards the bottom glass plate. The additional glass plate is placed on top of the parafilm, and weight (beaker filled with water) is placed on the upper plate to ensure the pressure of approximately 31.384 mmHg (**Fig S10-1**). The heater is set at 60°C, and the timer at 8 hours. Once heating is started the parafilm should be pressed down towards the lower glass plate to ensure optimal isolation and humidity during the transfer protocol. After 8 hours the heater is turned off and the beaker and the upper glass plate are removed carefully. Parafilm and 3 filter papers are removed with tweezers, and nitrocellulose is wetted with PBS, removed, and left to dry. Once dry the membrane can be analyzed with the standard NRP procedure. In order to enable NRP analysis of FFPE, several protocols were designed and tested and the protocol described above gave the best results both in the context of protein transfer (**Fig S10-2A**) and NRP results (**Fig S10-2B**). An image of NRP analysis of the membrane obtained with one protocol without enzymatic retrieval step and heating-facilitated transfer is shown in **Fig S10-2C** for comparison. Nevertheless, several questions remain to be answered and are in the focus of our ongoing research. First, we hypothesize that the heating protocol can oxidize the tissue samples, and therefore introduce error in the reductive capacity analysis of FFPE tissues by the NRP (or any other method focused on the assessment of FFPE tissue reductive capacity). Another possibility is that heat-induced protein denaturation/structural alteration may alter antioxidant function of the treansferred proteins. Robust and detailed experiments have to be designed in order to provide the answer to this question and help us understand the significance of heat-induced tissue oxidation in this context. Moreover, In the original protocol, we tested different enzymatic retrieval protocols, and different buffers, and good results were obtained by following deparaffinization and enzymatic retrieval protocol for proteomic expressional profiling of FFPE tissue by multiplex immunoblotting proposed by Chung and Hewitt [^22^](https://paperpile.com/c/54Y2KA/JBpr). Their protocol proposes the equilibration step in 50 mM ammonium bicarbonate buffer (pH 8.2) followed by treatment with a freshly prepared enzyme cocktail solution that contains 0.001% trypsin plus proteinase K in the same buffer. In their protocol protease inhibition step is added after the enzymatic retrieval and they propose a 15-minute long treatment with proBuffer (One tablet of complete protease inhibitor 0.5 ml of phosphatase inhibitor I and 0.5 ml of phosphatase inhibitor II in PBS (pH 7.2)) at room temperature. Nevertheless, in our preliminary experiments, enzymatic retrieval with greater concentrations of trypsin provided better results so we decided to follow this protocol. In regards to the protease inhibition step, our preliminary data suggest that results remain the same if this step is omitted so we didn’t place a protease inhibition step in our protocol although we initially planned. We hypothesize that proteases could still be at least partially inactivated, and heat could further facilitate this inactivation during the transfer protocol. As in our FFPE modification of HistoNRP, the heat-facilitated passive diffusion slice printing step follows the enzymatic retrieval, we consider this step to be optional based on our analyses. Nevertheless, we wanted to emphasize this as the end-user should be aware that protein degradation might take place during the protocol, and this might turn out to be important if the protocol is adapted or modified, and the tissue is not heated and transferred immediately. As briefly mentioned above, possible modifications and further understanding of this modified HistoNRP protocol for FFPE still remain an active area of research in our laboratory, so we believe further experiments might provide new information and result in further modifications of this protocol.


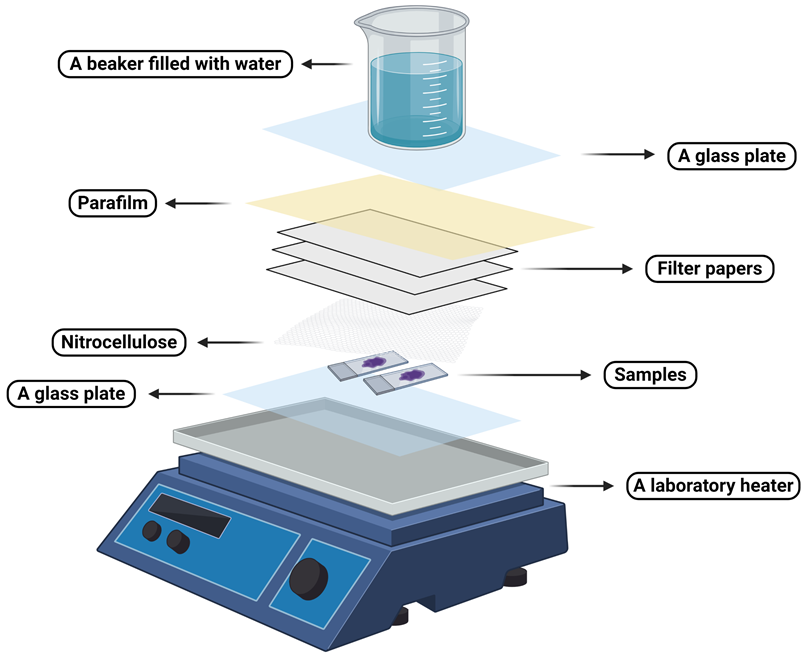

**Fig S10-1.** A schematic representation of the heat-facilitated passive diffusion slice printing setup.

**
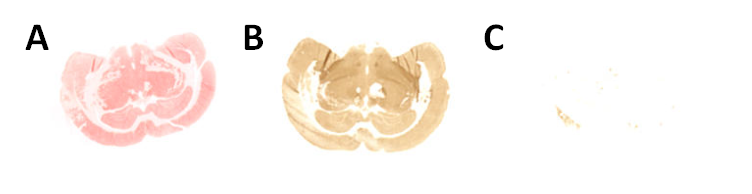
**

**Fig S10-2.** Analysis of FFPE brain tissue fixed onto the membrane by heat-facilitated passive diffusion slice printing. A) Ponceau S analysis of proteins transferred to the nitrocellulose membrane by heat-facilitated passive diffusion slice printing. B) An example of the FFPE brain tissue analyzed by NRP after heat-facilitated passive diffusion slice printing. C) An example of NRP analysis of the membrane obtained by passive diffusion slice print blotting from the standard NRP protocol instead of trypsin-based retrieval followed by heat-facilitated passive diffusion slice printing.

Supplementary material 11: A detailed explanation of the HistoNRP demonstration analysis illustrated in Fig 2 of the Main text

A coronal section of the rat brain-damaged unilaterally with the microdialysis probe was used for demonstration of the spatial distribution analysis of tissue reductive capacity illustrated in the Main text in Fig 2. A microdialysis experiment is explained in detail in Supplement 9. A standard HistoNRP protocol (as described in Supplement 1) was used for reduction capacity analysis and the membrane was digitalized both with an office scanner and with Samsung Galaxy S8. An image obtained by the Samsung Galaxy S8 camera was used for detailed analysis as it provided more anatomical details. Rat brain stereotaxic atlas was used to identify anatomical areas of interest [^23^](https://paperpile.com/c/MAtHtN/egrq) and Fiji software was used to calculate the distribution of pixel intensities in the inverted 8-bit image. In short, an image was imported into Fiji [File>Open…], converted to 8-bit [Image>Type>8-bit], and inverted [Edit>Invert]. First, the brain was divided by 6 lines of interest as shown in Fig 2H in the Main text and intensity profiles were calculated for both the ipsilateral and the contralateral side using the straight-line selection tool followed by the plot profile option [Analyze>Plot Profile]. Data was exported as a list, saved in an excel sheet, and imported in R software environment for statistical computing for further analysis and visualization with the ggplot2 package (Main text Fig 2I). Moreover, anatomical areas of interest were defined on the ipsilateral and contralateral side of the brain using the selection tool of the same size, and pixel intensity value distribution was calculated using the Histogram option [Analyze>Histogram ]. Values were exported as a list and saved in an excel sheet in the format of a list of pixel intensities with a corresponding number of pixels for the individual intensity value. Additionally, pixel distance was calculated for all areas of interest. Data were imported into R and visualized using the ggplot2 package (**Main text Fig 2J**).

References:
1. [Bioanalytical method validation - European Medicines Agency. *European Medicines Agency*](http://paperpile.com/b/1nYmMD/JWy6) <https://www.ema.europa.eu/en/bioanalytical-method-validation> [(2018).](http://paperpile.com/b/1nYmMD/JWy6)

2. [Lowry, O. H., Rosebrough, N. J., Farr, A. L. & Randall, R. J. Protein measurement with the Folin phenol reagent. *J. Biol. Chem.* 193, 265–275 (1951).](http://paperpile.com/b/iut3IQ/cp7A)

3. [Noble, E. P., Wurtman, R. J. & Axelrod, J. A simple and rapid method for injecting H3-norepinephrine into the lateral ventricle of the rat brain. *Life Sci.* **6**, 281–291 (1967).](http://paperpile.com/b/UfqKth/oEmy)

4. [Knezovic, A. *et al.* Staging of cognitive deficits and neuropathological and ultrastructural changes in streptozotocin-induced rat model of Alzheimer’s disease. *J. Neural Transm.* **122**, 577–592 (2015).](http://paperpile.com/b/UfqKth/rSyq)

5. [Grünblatt, E., Salkovic-Petrisic, M., Osmanovic, J., Riederer, P. & Hoyer, S. Brain insulin system dysfunction in streptozotocin intracerebroventricularly treated rats generates hyperphosphorylated tau protein. *J. Neurochem.* **101**, 757–770 (2007).](http://paperpile.com/b/UfqKth/fPZT)

6. [Salkovic-Petrisic, M. *et al.* Cerebral amyloid angiopathy in streptozotocin rat model of sporadic Alzheimer’s disease: a long-term follow up study. *J. Neural Transm.* **118**, 765–772 (2011).](http://paperpile.com/b/UfqKth/M1pV)

7. [Kliegman, R. M. & Morton, S. Sequential intrahepatic metabolic effects of enteric galactose alimentation in newborn rats. *Pediatr. Res.* **24**, 302–307 (1988).](http://paperpile.com/b/UfqKth/jCWY)

8. [Gage, G. J., Kipke, D. R. & Shain, W. Whole animal perfusion fixation for rodents. *J. Vis. Exp.* (2012) doi:](http://paperpile.com/b/UfqKth/rr7F)[10.3791/3564](http://dx.doi.org/10.3791/3564)[.](http://paperpile.com/b/UfqKth/rr7F)

9. [Lowry, O. H., Rosebrough, N. J., Farr, A. L. & Randall, R. J. Protein measurement with the Folin phenol reagent. *J. Biol. Chem.* **193**, 265–275 (1951).](http://paperpile.com/b/UfqKth/Nlvx)

10. [Oddo, S., Caccamo, A., Smith, I. F., Green, K. N. & LaFerla, F. M. A dynamic relationship between intracellular and extracellular pools of Abeta. *Am. J. Pathol.* **168**, 184–194 (2006).](http://paperpile.com/b/UfqKth/kku8)

11. [Lesné, S. *et al.* A specific amyloid-beta protein assembly in the brain impairs memory. *Nature* **440**, 352–357 (2006).](http://paperpile.com/b/UfqKth/FYk6)

12. [Vorhees, C. V. & Williams, M. T. Morris water maze: procedures for assessing spatial and related forms of learning and memory. *Nat. Protoc.* **1**, 848–858 (2006).](http://paperpile.com/b/UfqKth/51pi)

13. [Deacon, R. M. J. Assessing nest building in mice. *Nat. Protoc.* **1**, 1117–1119 (2006).](http://paperpile.com/b/UfqKth/yWPZ)

14. [Ayala, J. E. *et al.* Standard operating procedures for describing and performing metabolic tests of glucose homeostasis in mice. *Dis. Model. Mech.* **3**, 525–534 (2010).](http://paperpile.com/b/UfqKth/xApS)

15. [Walf, A. A. & Frye, C. A. The use of the elevated plus maze as an assay of anxiety-related behavior in rodents. *Nat. Protoc.* **2**, 322–328 (2007).](http://paperpile.com/b/UfqKth/TlVH)

16. [Knezovic, A. *et al.* Glucagon-like peptide-1 mediates effects of oral galactose in streptozotocin-induced rat model of sporadic Alzheimer’s disease. *Neuropharmacology* **135**, 48–62 (2018).](http://paperpile.com/b/UfqKth/0tTN)

17. [Babic Perhoc, A. *et al.* Cognitive, behavioral and metabolic effects of oral galactose treatment in the transgenic Tg2576 mice. *Neuropharmacology* **148**, 50–67 (2019).](http://paperpile.com/b/UfqKth/Skoy)

18. [Walters, G. C. & Abel, E. L. Passive avoidance learning in rats, mice, gerbils, and hamsters. *Psychon. Sci.* **22**, 269–270 (1971).](http://paperpile.com/b/UfqKth/A8HY)

19. [Seibenhener, M. L. & Wooten, M. C. Use of the Open Field Maze to measure locomotor and anxiety-like behavior in mice. *J. Vis. Exp.* e52434 (2015).](http://paperpile.com/b/UfqKth/kqrz)

20. [Bannur, S. V., Kulgod, S. V., Metkar, S. S., Mahajan, S. K. & Sainis, J. K. Protein determination by ponceau S using digital color image analysis of protein spots on nitrocellulose membranes. *Anal. Biochem.* **267**, 382–389 (1999).](http://paperpile.com/b/b7RRw4/bLeY)

21. [Sander, H., Wallace, S., Plouse, R., Tiwari, S. & Gomes, A. V. Ponceau S waste: Ponceau S staining for total protein normalization. *Anal. Biochem.* **575**, 44–53 (2019).](http://paperpile.com/b/b7RRw4/MHV1)

22. [Chung, J.-Y. & Hewitt, S. M. Proteomic expressional profiling of a paraffin-embedded tissue by multiplex tissue immunoblotting. *Methods Mol. Biol.* **1312**, 175–184 (2015).](http://paperpile.com/b/54Y2KA/JBpr)

23. [Paxinos, G. & Watson, C. *The Rat Brain in Stereotaxic Coordinates*. (Elsevier, 2005).](http://paperpile.com/b/MAtHtN/egrq)
